# Challenges in Elucidating the Free Energy Scheme of the Laccase Catalyzed Reduction of Oxygen

**DOI:** 10.1002/cctc.202200878

**Published:** 2022-12-07

**Authors:** Daan den Boer, Hendrik C. de Heer, Francesco Buda, Dennis G. H. Hetterscheid

**Affiliations:** ^1^ Leiden Institute of Chemistry Leiden University 2300RA Leiden The Netherlands

**Keywords:** laccase, oxygen reduction, free energy landscape, copper, catalysis

## Abstract

Artificial redox catalysts are typically limited by unfavorable scaling relations of reaction intermediates leading to a significant overpotential in multi‐electron redox reactions such as for example the oxygen reduction reaction (ORR). The multicopper oxidase laccase is able to catalyze the ORR in nature. In particular the high‐potential variants show a remarkably low overpotential for the ORR and apparently do not suffer from such unfavorable scaling relations. Although laccases are intensively studied, it is presently unknown why the overpotential for ORR is so low and a clear description regarding the thermodynamics of the catalytic cycle and the underlying design principles is lacking. In order to understand the laccase catalyzed ORR from an electrochemical perspective, elucidation of the free energy scheme would be of high value. This article reviews the energetics of the proposed laccase catalyzed ORR mechanisms based on experimental and computational studies. However, there are still remaining challenges to overcome to elucidate the free energy scheme of laccase. Obtaining thermodynamic data on intermediates is hard or even impossible with analytical techniques. On the other hand, several computational studies have been performed with significantly different parameters and conditions, thus making a direct comparison difficult. For these reasons, a consensus on a clear free energy scheme is still lacking. We anticipate that ultimately conquering these challenges will result in a better understanding of laccase catalyzed ORR and will allow for the design of low overpotential redox catalysts.

## Graphical abstract

**Bioinorganic chemistry**: High potential variants of the multicopper enzyme laccase have a remarkably low overpotential for the oxygen reduction reaction (ORR). Understanding the precise underlying principles are potentially of great value in light of the energy transition wherein the ORR plays a crucial role. The aim of this work is to review the progress that has been made in elucidating what makes laccases such excellent ORR catalysts, and to identify the remaining challenges in a complete elucidation of the free energy scheme for the ORR mediated by laccases.

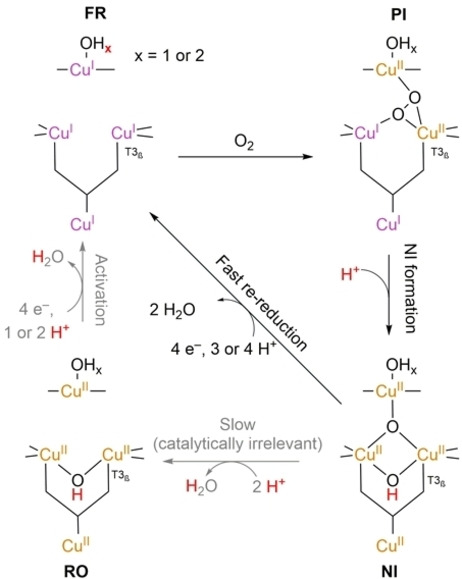

## 1 Introduction

### 1.1 Background information

**Scheme 1:**
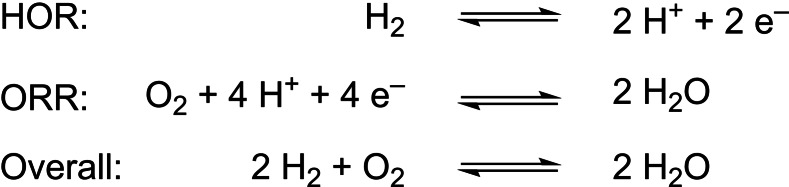
Combustion of H_2_ consisting of the anodic hydrogen oxidation reaction (HOR) and the cathodic oxygen reduction reaction (ORR). The thermodynamic standard potential is 1.23 V vs. NHE

The overexploitation of fossil fuels as energy source has given rise to environmental problems.^[1]^ The utilization of sustainable hydrogen obtained by water splitting is a good alternative as water is earth abundant and no greenhouse gasses are expelled. In the water splitting process, solar energy is stored in the form of chemical energy by the formation of hydrogen gas. Upon request, hydrogen can be transformed back to electricity by employing fuel cells, where the hydrogen oxidation reaction (HOR) takes place at the anode (Scheme 1).^[2]^ The protons and electrons produced react with molecular oxygen at the cathode, in the oxygen reduction reaction (ORR) yielding water. The equilibrium potentials of the HOR and ORR are 0.0 V and 1.23 V *vs*. the normal hydrogen electrode (NHE), respectively, under standard conditions (T=298.15 K, 1 atm O_2_ and H_2_ and 1 M [H^+^]), leading to an overall maximum cell potential of 1.23 V for the overall reaction.

However operating a fuel cell close to the theoretically maximum cell potential remains a challenge.^[3]^ Although the HOR is essentially reversible at a platinum electrode, the ORR proceeds only marginally at the equilibrium potential.[^3^, ^4^] Catalytic currents on the best noble metal electrodes only become significant at a ∼400 mV overpotential. Finding a more efficient and robust catalyst for the ORR is therefore of paramount importance for the utilization of renewable energy.

### 1.2 Heterogeneous catalysis

In general, catalysts enhance the reaction rate by effectively lowering the free energy of the transition state required for converting the reactants into products. Catalysts accomplish this by facilitating the formation of relatively stable intermediates whose formation is much more facile. Evidently, there is an optimum in the degree of intermediate stabilization, as the intermediates should remain reactive enough to transform into the desired product. A catalyst that binds the intermediate neither too strong, nor too weak is the best catalyst for the reaction in question. This is known as the Sabatier principle.^[5]^

In electrochemistry, the Sabatier principle is applied to reactions at or close to the thermodynamic equilibrium. The requirements for the best catalyst for a reaction at equilibrium can be made more specific: the best catalyst aligns reactants, products and all intermediates in terms of their free energy. This requirement derives from the fact that at their thermodynamic equilibrium, reactants and products have the same free Gibbs energy, as their formation are equally favorable at this potential. Any intermediate having another energy would invariably hamper the rate of interconversion, either by being more stable than the target product, or by providing a thermodynamic barrier to the interconversion process.

**Figure 1:**
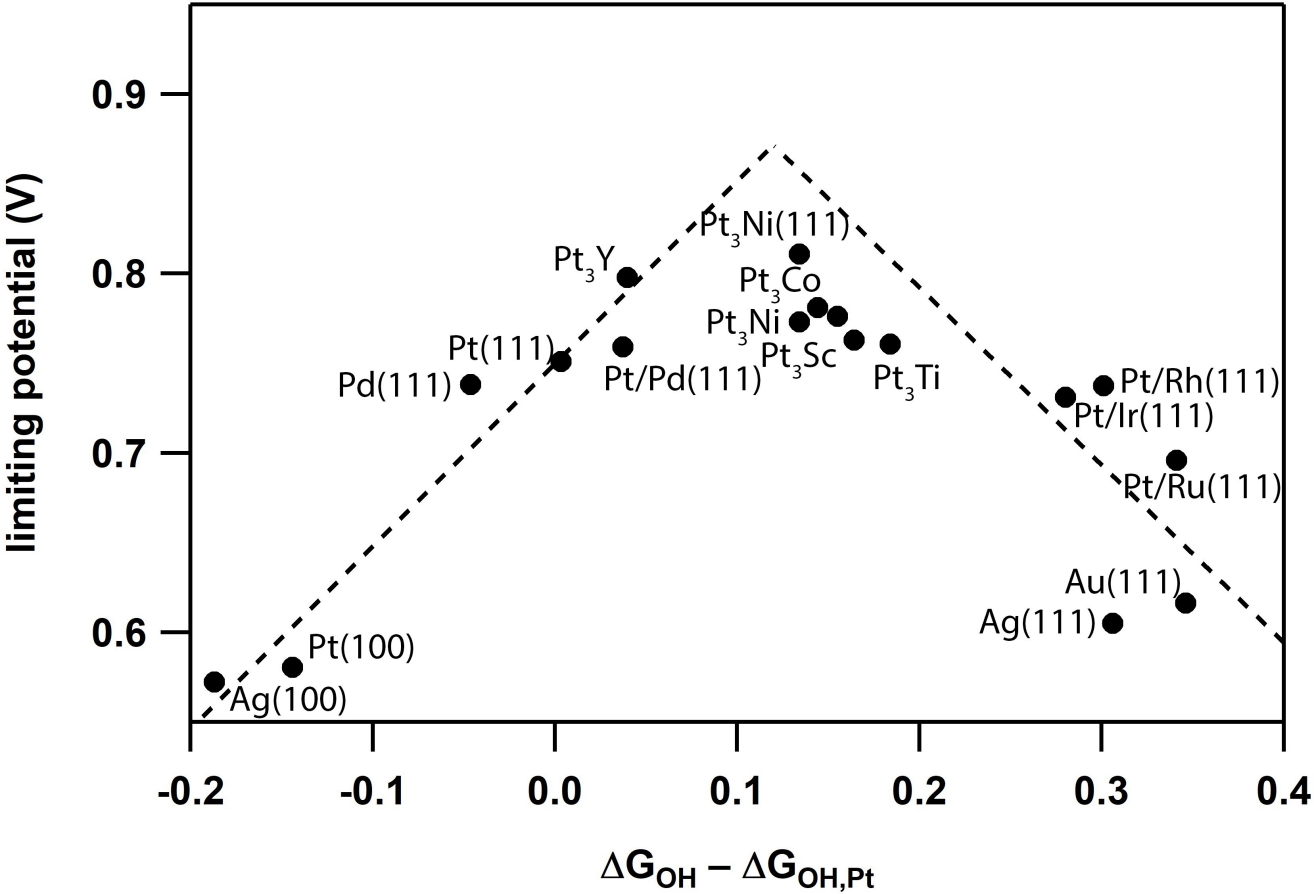
Volcano plot showing the experimental ORR potentials for a series of heterogeneous catalysts, showing that in the most optimized form the ORR reaction proceeds with a ∼400 mV overpotential. Data in the figure are based on literature.^[7b]^

The best known heterogeneous catalyst for the ORR is Pt, which operates at an overpotential of ∼400 mV (Figure 1).^[3]^ Despite extensive research in the last 15 years, limited successes are obtained in minimizing the overpotential for the ORR.^[6]^ Mechanistically, the Pt catalyzed ORR proceeds via three Pt bound intermediates, i. e. *OOH, *O and *OH, with different binding energies towards the Pt surface.^[7]^ Due to these binding energy differences, the Sabatier principle cannot be met for all intermediates simultaneously and consequently an additional overpotential is required to cycle through all catalytic intermediates. Thermodynamic optimization of these binding interactions is difficult as these intermediates are energetically related with each other in so called scaling relationships. In other words changing the binding energy for one intermediate will automatically change the binding energy for another intermediate as well.^[8]^ In the case of the ORR, a 1 : 1 scaling relationship exists for the *OOH and *OH intermediates, where the energy difference between these intermediates will always remain 3.2 eV, given that *OOH and *OH bind to Pt through the exact same interactions.^[9]^ Consequently, an ideal catalyst that meets the Sabatier principle and aligns all intermediates energetically is hard to realize. The most ideal compromise corresponds to a theoretic overpotential of roughly 400 mV.^[9]^

### 1.3 Bio‐electrocatalysis

In nature, the ORR is catalyzed efficiently by redox enzymes such as cytochrome *c* oxidases and multicopper oxidases (MCOs).^[10]^ The idea of immobilizing redox enzymes on electrodes was introduced in the early 1960s by Updike and Hicks, who called their system an *enzyme electrode*.^[11]^ ORR catalysis by means of enzyme electrodes was first investigated by Tarasevich et al. in the second half of the 1970s.^[12]^ Research on the catalytic activity for ORR of various immobilized oxidoreductases showed laccase to be a remarkably efficient catalyst for this reaction.^[13]^ Laccase immobilized on a carbon electrode is capable of catalyzing ORR through direct electron transfer (DET) with mass transport limiting catalytic currents at 100 mV overpotentials.^[14]^ The fact that laccase‐modified electrodes offer a significant gain in thermodynamic and kinetic efficiency relative to Pt electrodes has been confirmed in several recent publications: onset potentials of just 100 mV were obtained for ORR via direct electron transfer towards laccase on an anthracene‐modified Au electrode.^[15]^ Similar data was obtained for a laccase‐wiring redox hydrogel: at overpotentials of 200 mV,^1^ the laccase‐based cell outperformed a Pt electrode by 0.86 mA cm^−2^ vs. <0.35 mA cm^−2^.^[16]^ Moreover, the reversed reaction of ORR, i. e. the oxidation of water, can be catalyzed by laccase as well, showing that laccase must be a highly optimized catalyst that can operate close to the equilibrium potential of water.^[17]^

On a per‐site basis, laccase is much more active than Pt, even taking into account the fact that laccase contains 4 Cu ions (I=3 ⋅ 10^−25^ A/laccase enzyme *vs*. I=8 ⋅ 10^−27^ A/Pt surface atom).[^12^, ^18^] Apparently, ORR catalysis in nature proceeds via a pathway with scaling relationships that are more favorable than those of the heterogeneous catalysts that are currently available. Here it is important to mention that the redox potentials of most MCOs are too low to operate close to the equilibrium potential of water, and that the features discussed above are limited to a very select group of high potential laccases.

### 1.4 Scope of this review

Conceptually laccase operated fuel cells are very interesting as these can operate at low overpotentials and can circumvent the application of expensive materials.^[19]^ Although laccase is one of the most studied oxidoreductases, and applied in for example lignin removaL, textiles and fibers, bioremediation, food and beverages, detergents and paints,^[20]^ it is presently unclear why the overpotential of ORR catalyzed by laccase is so remarkably low. In order to develop an understanding for this behavior from an electrochemical perspective, a free energy scheme (FES) with respect to a standard reference electrode is indispensable. In an earlier publication, a FES for cytochrome c oxidases could be constructed using a combination of mechanistic and thermodynamic data derived both from experiment and from simulations. This was of great explanatory value for the overpotentials at which cytochrome c oxidases operate.^[10a]^ The aim of this work is to review the progress that has been made in unraveling the FES, and to identify the remaining challenges in elucidating the FES by an assessment of experimental and computational data of the ORR mediated by laccases. Before further addressing this objective, the physiological role and structure of laccase will be discussed with a focus on the active site.

## 2 Discovery, function and structure of laccase

### 2.1 Discovery and etymology

Laccases are a class of multicopper oxidoreductases or, equivalently, multicopper oxidases (MCO) enzymes, named so for their copper‐based active site.[^11a^, ^21^] The first isolated low‐potential laccase was reported in 1883 by the Japanese scientist Yoshida, who obtained it from the secretion of *Rhus vernicifera* (*Rv*L), the Japanese lac tree.^[22]^ On top of that, Yoshida demonstrated laccases capability to oxidize a substrate present in the tree's secretion in the presence of oxygen in a moist environment. In the 1890s, Bertrand called the enzyme laccase, a name obviously derived from its presence in the lac tree. Bertrand found the enzyme to be present also in gum Arabic and gum SenegaL, as well as in numerous plants and fungi.^[23]^

### 2.2 Occurrence, physiological role and substrates

As it is apparent from Bertrand's studies, laccase is not only found in the Japanese lac tree. Although laccase derived from *Rhus vernicifera* is the most extensively studied of all laccases, its occurrence in higher plants is limited.[^21^, ^24^] It is found mostly in fungi and (lower) plants, but presence in various insects and prokaryotes has also been reported.[^11a^, ^21a^, ^24a^, ^25^] Concerning their physiological role, laccases are employed for a wide variety of purposes. In plants, they catalyze lignification and wound healing.[^24b^, ^25^] In fungal laccases, they also play a role in morphogenesis, pigment formation, lignin detoxification and degradation, and protection of fungal pathogens against host immunoresponse.[^21a^, ^25^] Plant and fungal laccases catalyze the oxidation of a broad range of substrates.[^21^, ^25^] *Rv*L for instance naturally catalyzes the oxidative oligomerization and polymerization of catechol derivatives in which the meta‐ or para‐positions are substituted by penta/heptadecadi‐ and/or trienyl chains.^[26]^ Next to these, the enzyme catalyzes oxidation of dopamine, quinoL, 2,2′‐azino‐bis(3‐ethylbenzothiazolinesulfuric acid), (ABTS), syringaldazine, inorganic substrates such as ferrocene monocarboxylic acid (FMCA), carbon dioxide and pentaerythrityl radical and ferricyanide.^[27]^ The enzyme facilitates all these oxidations by concomitantly reducing O_2_ to H_2_O, the reaction of interest in this work.[^21^, ^28^]

### 2.3 Structure of laccase

#### 2.3.1 Overall structure of laccases

**Figure 2:**
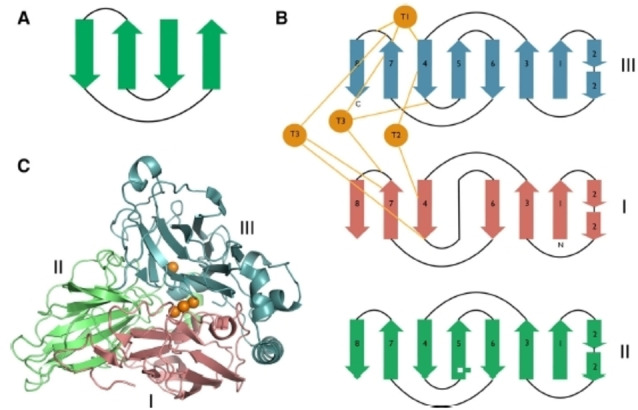
Secondary, tertiary and quaternary structure of 3D laccases. A) The Greek‐key shape of the cupredoxin β‐barrel motif. B) Secondary structure of the three domains. Ligation to the three types of Cu ions (shown in orange) is facilitated by residues embedded in domains I and III. C) Tertiary structure of laccase (from Melanocarpus Albomyces): the three domains fold around the 4 Cu ions to create the T1 site and the TNC site separated by 12–13 Å. Regarding the quaternary structure, three‐domain laccases are monomers. Reproduced from Ref. [25]. Copyright 2015, with permission from Springer Nature.

In generaL, laccases can be categorized in two subgroups, based on the number of domains of which they consist. Up till 2009, only X‐ray structures of three‐domain (3D) laccases were reported. Later, also a number of two‐domain (2D) laccases were identified.^[28]^ This work will only focus on the 3D laccases and simply refer to these as *laccases*, since the 2D laccases have not been subject to such extensive research as their 3D counterparts. More importantly, in 2D laccases the active site in which ORR takes place differs in several fundamental aspects from the canonical active site as found in 3D laccases.^[29]^ In terms of their primary structures, laccases derived from different organisms have been found to differ strongly from one another.[^24c^, ^28^, ^30^] For instance, in a comparison of the amino acid sequences in *Rv*L and laccase from *Trametes versicolor* (*Tv*L) several dozen residues were found to be mutually different.^[30c]^ Nonetheless, the overall structures of different laccases show remarkable similarities. This especially concerns their active site geometry, the first coordination sphere and some common 2^nd^ coordination sphere residues.[^21^, ^28^, ^31^] Regarding their secondary structures, laccases are characterized by three domains, which are all folded in the so‐called cupredoxin fold, which is built up from several sequential β‐sheets aligned in an antiparallel fashion forming a closed structure known as the Greek‐key barrel (Figure 2A and Figure 2B).[^11a^, ^28^] The three domains together form the tertiary structure, offering a scaffold for two separated, interconnected copper centers, one containing a single Cu ion, the other site containing three Cu ions (Figure 2C).

**Figure 3:**
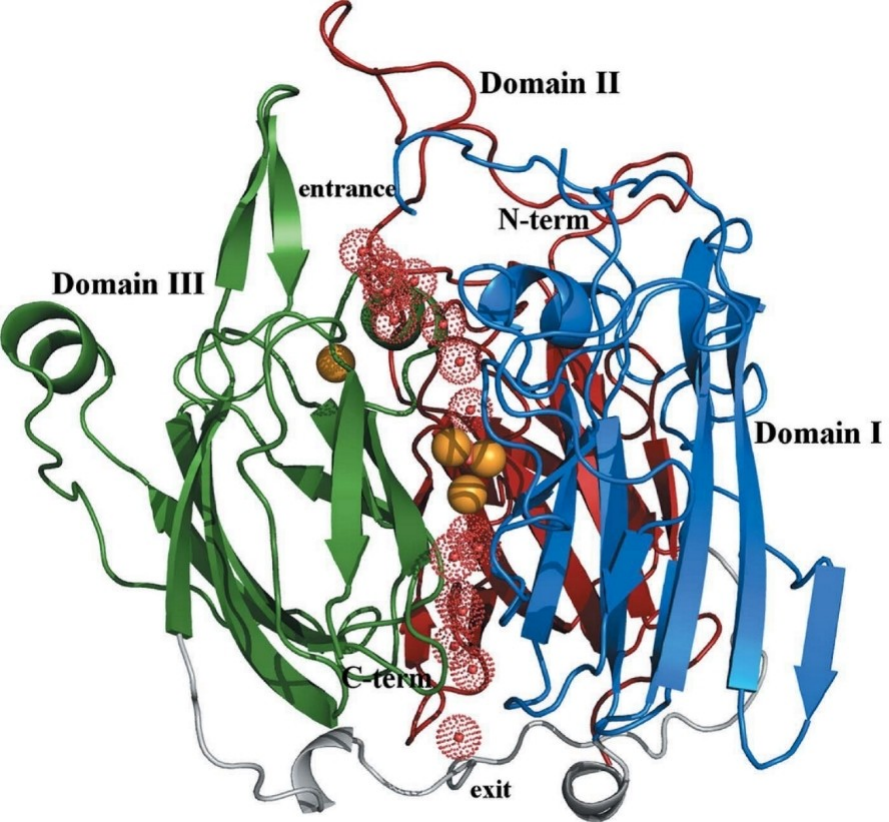
Hydrophilic tunnels highlighted by the perforated red spheres, which represent water molecules, leading towards and away from the TNC (indicated with the three orange spheres, corresponding to the three Cu ions). The tunnels are located on the interface between the three domains. Reproduced from Ref. [34d]. Copyright 2012, with permission from IUCr journals.

The site with the single Cu ion is incorporated in a cavity at the molecular surface of the enzyme in domain III.[^30a^, ^30b^, ^32^] At this site substrates are oxidized.[^21b^, ^28^] The other site, known as the trinuclear cluster (TNC), is buried 12–13 Å towards the interior of the enzyme, and is accessible from the surface by two hydrophilic tunnels, depicted with the perforated red spheres in Figure 3.[^21^, ^28^, ^32b^, ^33^] The ORR is catalyzed inside the TNC pocket.^[21]^ Reactant protons and the H_2_O produced in this reaction are probably transported via these hydrophilic tunnels.[^30a^, ^32a^, ^34^] The diffusional path of O_2_ itself towards the catalytic pocket is still subject of debate.[^30b^, ^34^, ^35^]

#### 2.3.2 Structure of the active site

##### 2.3.2.1 First coordination sphere

**Figure 4:**
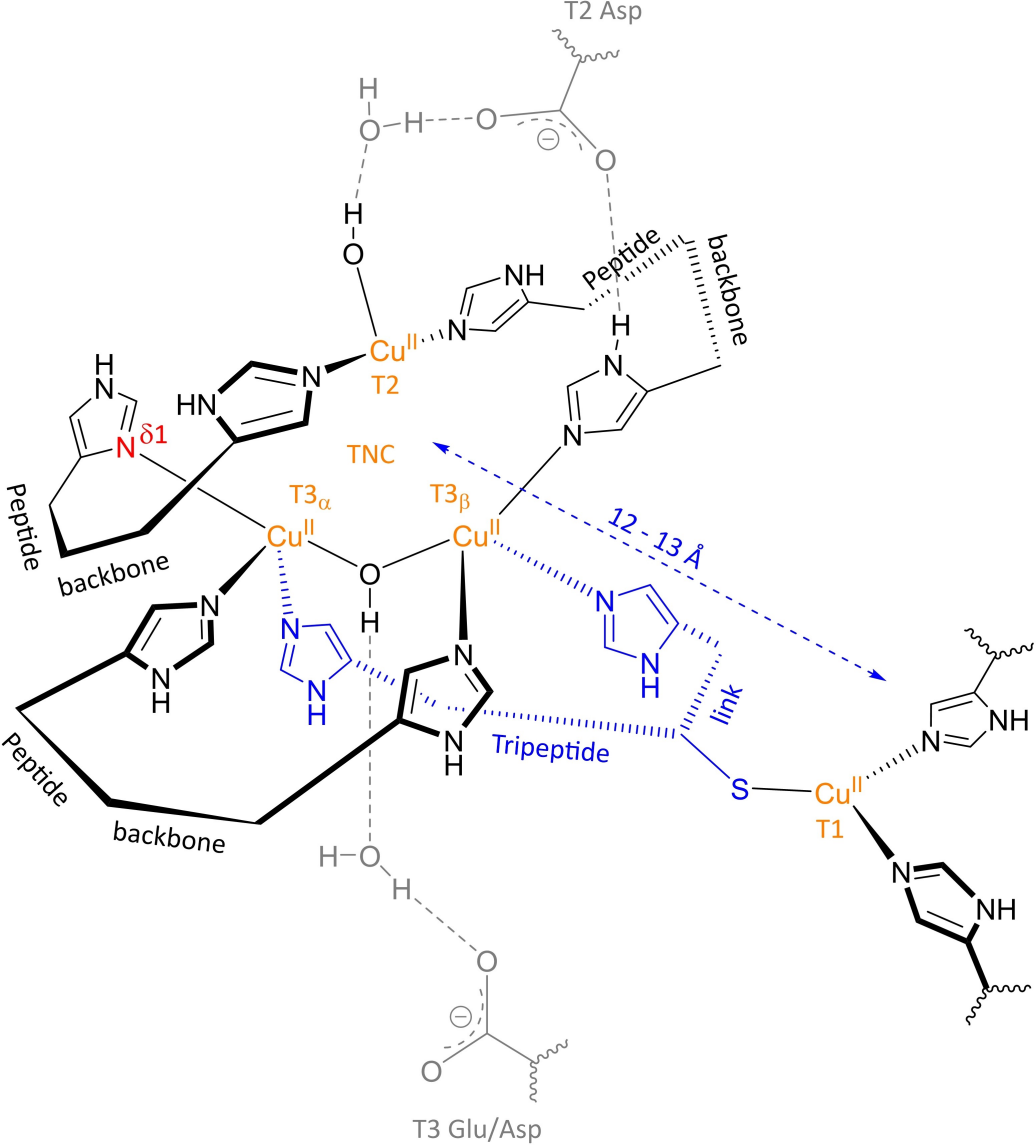
Schematic representation of the copper sites in (3D) laccase. Orange: T1 Cu^II^ center and TNC containing two T3 and a T2 Cu^II^ ions. Black: protein backbone with explicitly depicted imidazole functionalities of the His residues directly coordinated to the Cu^II^ ions. Blue: bifurcated His‐Cys‐His link connecting T1 and TNC, covering a distance of 12–13 Å. Note the non‐identical geometry of T3_α_ and T3_β_: A trigonal bipyramidal geometry of T3_α_ is obtained by δ1 N coordination of the axial His ligand, highlighted in red. T3_β_ is a distorted trigonal bipyramid due to ϵ2 N coordination of all His ligands. Grey: 2^nd^ coordination sphere residues which have been shown to be vital for ORR catalysis. This figure is based on Heppner et al. 2014.^[37]^

As already outlined above, laccases are characterized by two distinct copper sites. The first site containing a single Cu ion and the other with three Cu ions (TNC) are located 12–13 Å apart (see Figure 4). In the enzyme's resting state, these are all Cu^II^ ions. Because the Cu^II^ ions have well defined spectroscopic features, they are indicated by distinct labels: in the first place, there is a type 1 (T1) Cu^II^, also known as *blue* Cu^II^ due to its strong absorbance in the visible (VIS) range at ∼600 nm (ε ∼5000 M^−1^ cm^−1^).^[33]^ Secondly, there is a type 2 (T2) Cu^II^, which has no significant VIS absorption, but only a weak d→d transition at ∼600 nm (ε ∼40 M^−1^ cm^−1^) associated with a tetragonal complex.^[36]^ Next to the T1 and T2 Cu^II^ ions, laccases contain two spectroscopically *coupled* type 3 (T3) Cu^II^ ions. The coupled T3 Cu^II^ ions are defined by a broad absorption band close to the ultraviolet (UV) range at 330 nm (ε=2800 M^−1^ cm^−1^).^[33]^ In Figure 4, the distribution of the Cu^II^ ions over both sites is highlighted in orange. Figure 4 also displays the first coordination sphere of the Cu^II^ ions and two second coordination sphere carboxylate moieties which have been reported to be of vital importance for the enzyme's capacity to catalyze ORR.^[21b]^

The single Cu electron transfer site contains the T1 Cu^II^ ion, and the T2 and T3 Cu^II^ ions are embedded in a triangular fashion in the TNC.[^21^, ^30a^, ^32a^, ^33^] The 12–13 Å gap between the T1 site and the TNC is covered by a bifurcated tripeptide link consisting of a histidine (His) followed by a cysteine and another His (His‐Cys‐His link, indicated in blue), of which the Cys coordinates to T1 Cu^II^, and each of the two His residues to one of the T3 coppers.[^21^, ^28^] In fungal laccases, T1 Cu^II^ has two His ligands next to the Cys ligand from the tripeptide link, yielding a trigonal planar complex, as shown here.[^11a^, ^21b^, ^30a^] Plant laccases have a fourth ligand coordinated to the T1 Cu^II^, the resultant geometry being a trigonally elongated tetrahedron or a distorted trigonal bipyramid if the open coordination site is taken into account.[^21b^, ^30a^]

Regarding the TNC, the T3 coppers each have three axially coordinated His ligands, of which one is a component of the tripeptide link. In the resting oxidized (**RO**) state, the T3 coppers are connected by a μ‐OH^−^ bridge.[^30a^, ^33^, ^38^] Both complexes have a trigonal bipyramidal geometry, each with an open coordination site directed into the TNC. In one of the T3 complexes however, this geometry is distorted. The Cu^II^ ion in the complex with the distorted geometry is labeled as T3_β_ in order to distinguish it from the T3 Cu^II^ ion in the complex with intact geometry, which is tagged T3_α_ Cu^II^.^[39]^ The geometry distortion in the T3_β_ complex is a consequence of not all His ligands coordinating towards the T3 coppers with the same N‐atom, as was shown by computational studies.^[39]^ In case of the T3_α_ one His is coordinated with its δ1 N (highlighted in red), the other two with their ϵ2 N atoms. The T3_β_ complex only has ϵ2 N coordinated His residues. Consequently, T3_β_ Cu^II^ is tilted out of the plane formed by the His ligands by 0.2–0.3 Å.^[40]^ In 2015, Hakulinen and Rouvinen analyzed 90 laccase crystal structures available in the protein data bank (PDB), and found this feature to be present in all of them.^[28]^ In some more recently reported crystal structures of two thermostable laccases, the same geometric features of the two T3 coordination geometries were found as well.^[32b]^

The T2 complex has two His ligands, and a water‐derived (WD) ligand. Together with an open coordination site directed into the TNC, these ligands are arranged in a square planar geometry around the T2 Cu^II^ ion.[^11a^, ^21^, ^33^, ^41^] In *Rv*L, the WD ligand has been found to be a OH^−^ moiety in the resting state of the enzyme for pH values ranging from 4.7 to 10.^[41]^

##### 2.3.2.2 Important second coordination sphere features

Laccases also have a set of comparable features in the second coordination sphere. Some of these residues close to the TNC (∼8 Å) are especially worth mentioning, as they are pivotal in both active site morphology and the ORR mechanism.[^31^, ^42^] These residues and their H‐bound connectivity to the first coordination sphere are included in grey in Figure 4. In the first place, this concerns a deprotonated aspartate (Asp) residue located at the entrance of one of the hydrophilic tunnels in the vicinity of T2 and T3_β_ Cu^II^, labeled as T2 Asp in Figure 4.[^34f^, ^38^, ^39^] This residue provides an H‐bonding network to the T2/T3_β_ edge of the TNC, which is critical for the T2 and T3_β_ structure.[^31a^, ^38^] Furthermore, it stabilizes the coordination unsaturation in the TNC and tunes the redox properties of the T2 and T3 coppers.[^38^, ^39^, ^41^] It is also involved as a proton shuttling group in the ORR cycle.^[31a]^ Next to T2 Asp, Figure 4 shows a second important carboxylic functionality, originating from a glutamate (Glu) or Asp residue in the proximity of the T3 Cu^II^ ions and one of the hydrophilic tunnels, referred to as T3 Glu/Asp. This residue is positioned also in the hydrophilic channel and is located at 6–7 Å from the T3 center opposite to T2 Asp.[^31b^, ^34f^] It is hydrogen bonded to the μ‐OH^−^ bridge via crystalline water.^[31b]^ Spectroscopic and simulation studies on (mutant) MCOs have revealed that it is involved in the ORR mechanism as a proton donor, although it does not significantly affect the structure of the TNC.[^38^, ^42^]

### 2.4 ORR catalysis at the TNC

**Scheme 2:**
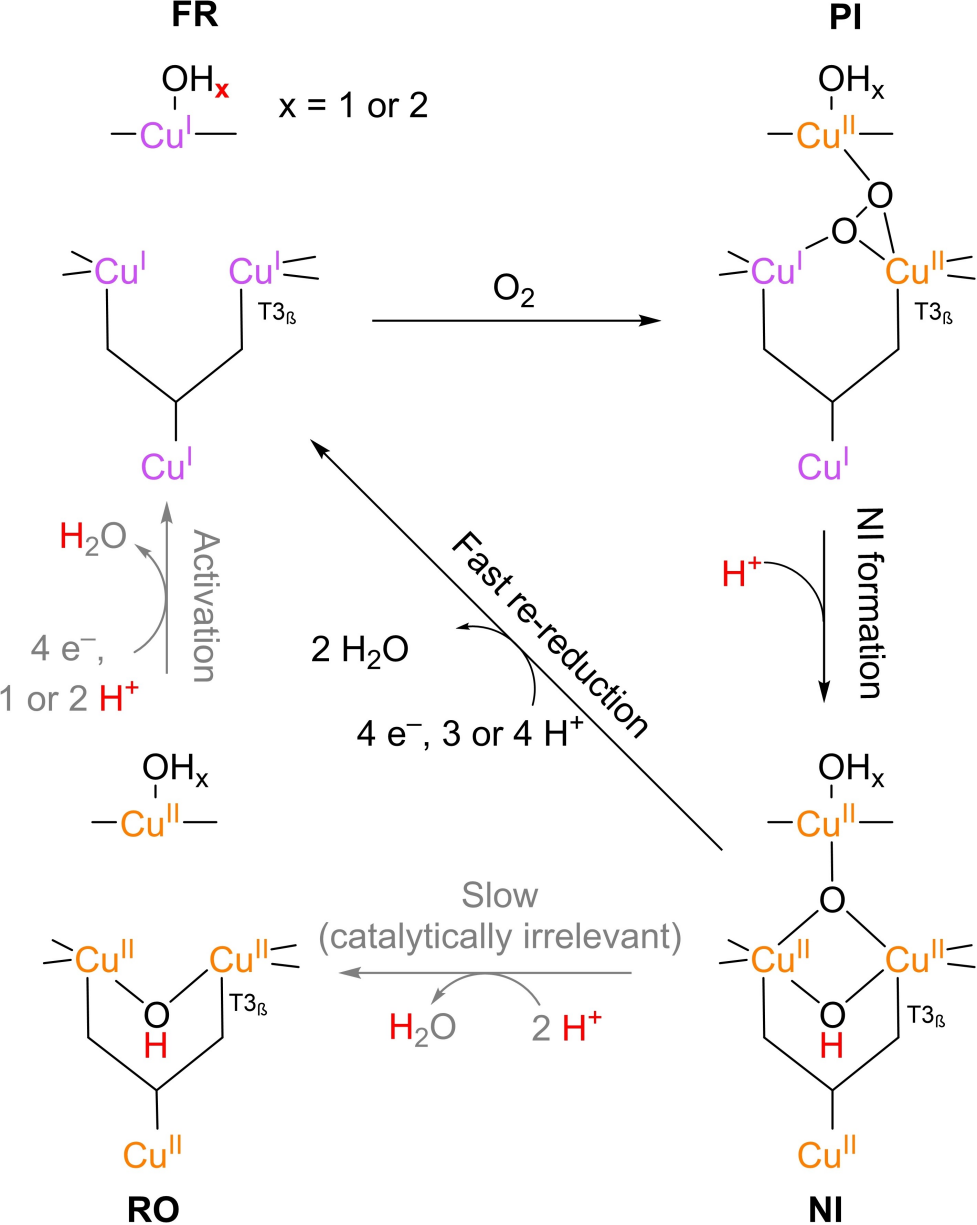
General catalytic cycle for ORR in the TNC. His/Cys ligands are shown in a skeleton‐like fashion for brevity. Arrows indicated in grey are catalytically less relevant. The reaction of the resting oxidized (**RO**) to the fully reduced (**FR**) state is an activation step. The actual ORR cycle starts when O_2_ diffuses into the TNC to form the peroxide intermediate (**PI**). **PI** quickly decays to the native intermediate (**NI**) in 2 double IET steps, in which a proton is transferred most likely from the carboxylic acid moiety of the T3 Glu/Asp residue. **NI** has the same oxidation state as **RO**. The ORR cycle is closed by the proton‐coupled re‐reduction of the **NI**, consuming four equivalents of oxidizing substrate under excretion of 2 equivalents of water. **NI** to **RO** is a deactivation step, which becomes significant only when no oxidizing substrate and/or O_2_ is present anymore. This mechanism is based on a catalytic cycle provided by Jones and Solomon 2015.^[21b]^

As for the catalytic pathway, along which ORR proceeds in the TNC, the present insights allow for a general cycle with multiple key reaction intermediates (Scheme 2). The cycle is general in the sense that it does not reveal the intermediates involved in the elementary reactive steps associated with the conversion of one key intermediate into the next. Currently, there is consensus on the order in which the key intermediates occur in the general catalytic cycle. Yet the mechanisms involved in the sequential conversion of the key intermediates are still subject of debate.[^21b^, ^43^] The various insights in the exact intermediate structures as well as the mechanistic and thermodynamic details of their formation will be dealt with in section 3.

The catalytic ORR cycle starts with the complete reduction of all Cu^II^ ions in the resting oxidized (**RO**) state to form the fully reduced (**FR**) state of the enzyme. This reaction is proton‐coupled and water is excreted from the enzyme. After this activation step, O_2_ binds to the TNC to form the peroxide intermediate (**PI**). In this process, internal electron transfer (IET) takes place from two of the TNC Cu^I^ ions to the coordinating O_2_. The O−O bond in the peroxide ligand is subsequently broken in a proton‐coupled IET process. The resulting intermediate is referred to as the native intermediate (**NI**), since it is the only intermediate that has actually been observed in any WT MCO catalyzing ORR. The Cu^II^ ions in the **NI** have the same oxidation state as in the **RO** state. The ORR cycle is closed by a re‐reduction reaction in which the **NI** state is converted into the **FR** state by sequential proton‐coupled reduction of the Cu^II^ ions under excretion of two H_2_O molecules. The enzyme is then ready to reduce another O_2_ molecule. In case of full depletion of O_2_, the **NI** slowly decays to the **RO** state. In case of the ferroxidase MCO enzymes Ceruloplasmin and Fet3p **NI** decay has been reported to proceed via **RO** in the catalytic cycle.^[44]^ For most other MCOs this process is catalytically irrelevant. It will therefore not be discussed in detail.

The general mechanism or ORR is primarily obtained from a variety of analytical and simulation tools applied to a small number of specific MCOs, such as iron transport protein 3 (Fet3p) and *Rv*L. As all the MCOs have remarkably similar active sites, the insights into the catalytic functioning of those species that have actually been investigated is expected to be applicable to the entire range of similar MCOs.^[21b]^ Although the **RO**, **FR**, **PI** and **NI** states are common intermediates through which all laccases turnover, it depends on the type of laccase whether IET or activation of O_2_ is rate limiting. For laccases with a low potential T1 site IET is fast, while it becomes rate limiting in case of laccases with high potential T1 sites.[^30c^, ^44a^, ^45^] See section 3.2.2. for a detailed discussion on low potential and high potential laccases.

The most important methodology in elucidating the catalytic intermediates in the general cycle discussed above was to modify or deplete the T1 and T2 copper centers. In the first case, the T1 Cu^II^ is substituted by Hg^II^, a redox‐innocent and spectroscopically silent cation. The resulting mutant laccase is referred to as T1HgLc. In the second case, Cu loading at the T1 or T2 site is inhibited by site selective mutations in the enzyme's genetic code to yield T1 or T2 depleted (T1D or T2D) MCOs.^[21b]^ In such mutant MCOs, the catalytic cycle is essentially ‘crippled’, and consequently stagnates on its way to produce water, enabling one to trap otherwise undetectable intermediates. Under the well supported assumption that the part of the mechanism until stagnation has remained intact as compared to the mechanism in WT laccase, the general catalytic cycle could be resolved using a variety of analytical and computational techniques.[^21b^, ^38^, ^46^] The conclusions drawn from these studies for each specific transformation in the cycle will be discussed in section 3.

### 2.5 Precedence for the alternative resting state AR

**Figure 5:**
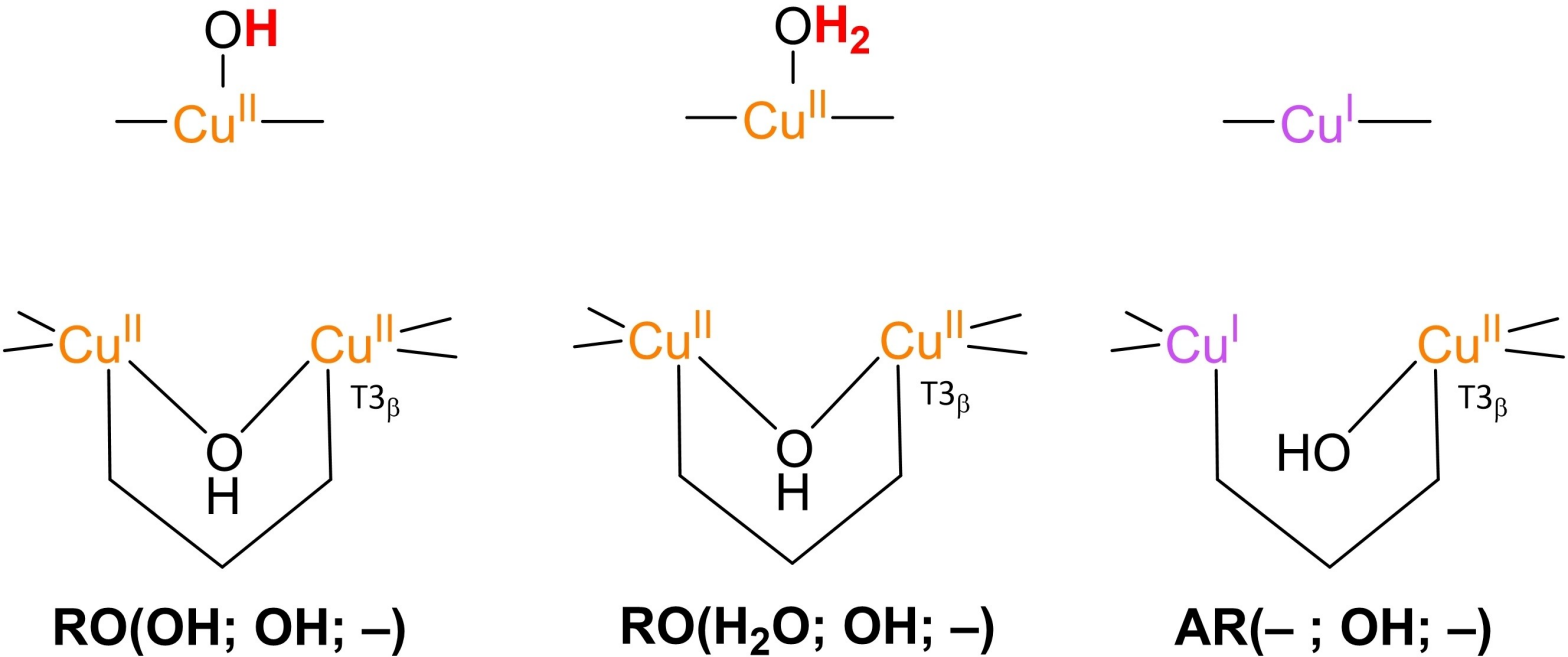
Comparison of **RO**(OH; OH; –), **RO**(H_2_O; OH; –) and **AR**(–; OH; –). The structure **RO**(OH; OH; –) was found to be more stable than **RO**(H_2_O; OH; –).

An alternative resting state to **RO** has been proposed recently for the high‐potential bilirubin oxidase *magnaporthe oryzae* (*Ma*BOD) and the high‐potential laccase P. anserina (*Pa*L).^[47]^ In the alternative resting (**AR**) state only one of the three Cu centers in the TNC is oxidized in comparison with the **RO** state in which all Cu centers are oxidized (see Figure 5). Despite partly reduction of the TNC, the **AR** form was found unable to perform the two electron reduction of O_2_.^[47b]^ Thus far, the **AR** state has not been observed in case of any low‐potential laccases or even other laccases besides PaL. However, it is important to note that spectroscopic distinction between the **RO** and **AR** states is not that straightforward as both the T3 sites in **AR** and in **RO** are EPR silent. For **AR** a g_∥_=2.32–2.27 and A_∥_=79 ⋅ 10^−4^ cm^−1^ is found in EPR measurements corresponding to the T2 site, while values of *g*_∥_ of 2.22–2.27 and *A*_∥_ of |170–200|×10^−4^ cm^−1^ are typical for **RO**. In contrast to **RO**, no 330 nm charge transfer band is present for **AR**.^[47b]^

Thus far potentiometric data for the **AR** state is not available and the species has not been included in any computations. It is therefore not discussed in great detail in the next section.

## 3 Mechanistic and thermodynamic details of ORR catalysis in laccase

### 3.1 Nomenclature for referring to intermediates

In the following section, relevant key intermediates will be referred to by the codes as introduced in Scheme 2, followed by a specification between brackets on the nature of the T2 WD‐ligand, the T3 bridging ligand (if any), and the TNC central ligand (if any) respectively. The formal charge of those ligands is omitted in this nomenclature. For example, the **RO** state as depicted in Scheme 2 will be referred to as **RO**(OH, OH, –). A **PI** state with an OH^−^ as T2 WD‐ligand and a centrally coordinated O_2_^2−^ ligand in μ_3_‐1,1,2‐fashion is referred to as **PI**(OH; –; μ_3_‐1,1,2‐O_2_), etc. If a key intermediate is reduced further by one or more electrons over the course of catalysis, this will be indicated with a (**‘**) symbol per electron (e. g. **PI’**(H_2_O; μ_3_‐O_2_; –)).

### 3.2 Enzyme activation: from RO to FR

#### 3.2.1 Mechanistic details

Before the enzyme is ready to perform any catalytic ORR cycle, the **RO** is fully anaerobically reduced by sequential oxidation of four substrates at the T1 Cu. This is the activation step depicted in Scheme 2. If the enzyme is immobilized on an electrode surface, substrate oxidation is replaced by a DET process from that surface to the TNC of the immobilized laccase. The TNC Cu^II^ ions are normally reduced by IET via the His‐Cys‐His link after reduction of the T1 Cu^II^ by reducing substrate or by DET from the electrode surface to T1 Cu^II^.[^21^, ^27c^, ^27e^, ^27f^, ^32a^, ^48^] In this process, the μ‐OH^−^ bridge in the **RO** state is protonated and excreted as H_2_O.^[21b]^ This is primarily concluded based on a crystal structure of the **FR** state of the MCO ascorbate oxidase (AO), in which the μ‐OH^−^ ligand was absent.^[49]^ Absence of this ligand in the **FR** state was later confirmed in simulation studies as well.^[31b]^ Moreover, it was recognized in early experiments on *Rv*L that the reduction kinetics of the two T3 Cu^II^ ions is proton dependent, as would be expected for proton loss of the μ‐OH^−^ ligand.[^27b^, ^48c^] The pK_a_ of the involved proton transferring group was estimated to be ∼7.4 in *Rv*L (Possibly an overestimation due to a wrong mechanism suggestion), and ∼3.5 in *Trametes versicolor* laccase (*Pv*L).^[50]^ Initially, the proton‐dependence was not associated with proton‐coupled dissociation of the μ‐OH^−^ bridge, but deemed to originate from a protonation event of a T2 OH^−^ ligand to form H_2_O.^[50]^ This conclusion was strengthened by electron paramagnetic resonance (EPR) studies on the T2 site in the **RO** state of a T1HgLc mutant of *Rv*L. Tamilarasan and McMillin claimed T1HgLc to show pH‐dependence in the EPR spectrum, with a pK_a_ between 6 and 7. Although the observed changes in the spectra were very small, the authors claimed they were significant enough to derive a pH‐dependence from them. The pH‐dependence was attributed to deprotonation of a T2 H_2_O ligand, yet they kept the option open of deprotonation of another group in the periphery of the T2 site.^[51]^

**Scheme 3:**
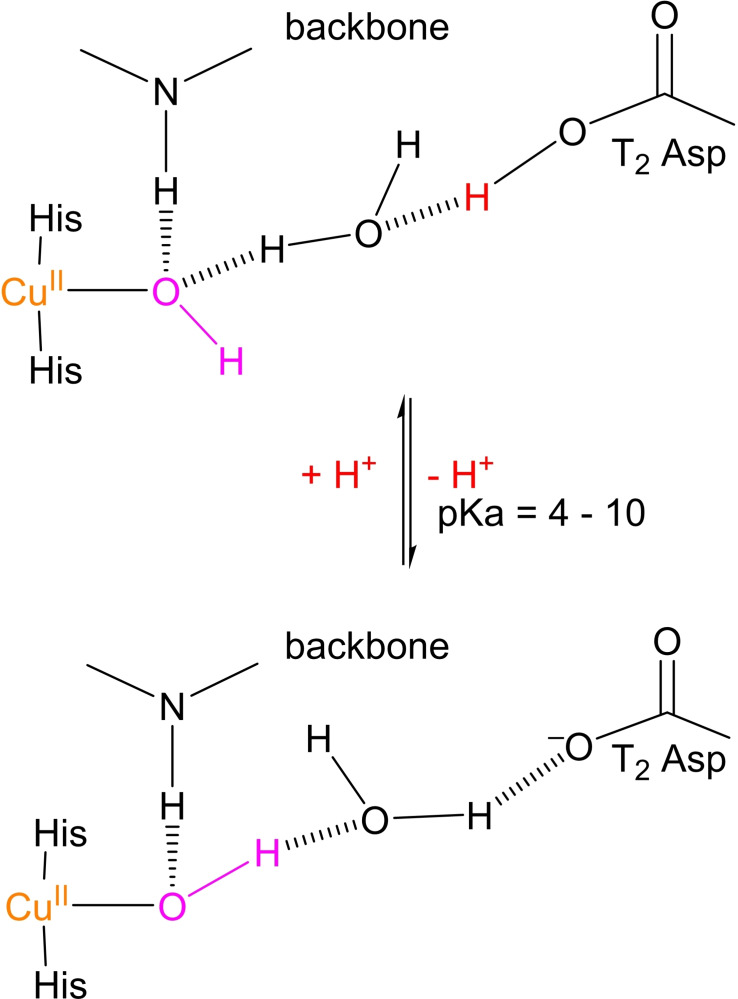
The pH effect of the spectroscopic (MCD) features of the T2 site in the RO state of the MCO is explained in terms of configurational changes in the H‐bond network of the T2 Cu^II^ ion's 2^nd^ coordination sphere. The T2 WD ligand (depicted in magenta) remains OH^−^ throughout the proton‐exchange process, yet its coordination geometry is significantly affected by deprotonation (highlighted in red) of the carboxylic acid moiety of T2 Asp. The effect was considered independent of the T3 Glu/Asp residue as mutants of that residue to an amino acid without a carboxylic moiety hardly affected the T2 spectroscopic signals.

Contrary to the conclusions of Tamilarasan and McMillin, Quintanar et al. reported the multifrequency EPR spectrum of the **RO** state of the same mutant enzyme (T1HgLc of *Rv*L) to be pH independent between pH 4.7 and 7.5.^[41]^ Furthermore, they performed magnetic circular dichroism (MCD) studies at various deuteron concentrations, which showed a pD‐dependent absorption band with an associated pK_a_ of 5.5±0.8. Analysis of the other features of the spectrum led the authors to rule out significant perturbations of either the T2 ligands or the coordination number of the T2 Cu^II^. Based on these and other observations, they concluded the T2 WD‐ligand in the **RO** state of T1HgLc to be OH^−^ between pH 4 and 10. The MCD absorption band with an associated pK_a_ of 5.5±0.8 was assigned to deprotonation of the T2 Asp residue with concomitant conformational changes in the first coordination sphere of the T2 Cu^II^, as shown in red and magenta respectively in Scheme 3.^[41]^ This assignment is further backed by mutation studies on T1 Cu^II^ depleted (T1D) Fet3p.[^31a^, ^42^] In this mutant MCO, the same pH‐independence of the EPR spectrum was observed as in the T1HgLc mutant. The spectroscopic analogy between both enzymes also held regarding the pD‐dependent absorption band in the MCD spectrum.^[31a]^ Spectroscopic analysis of T1D Fet3p in which the T2 Asp and/or T3 Glu residue were substituted by other amino acids showed that the T3 Glu residue is not involved in the pH‐effect of the MCD spectrum of the T2 site in the **RO** state of Fet3p, but T2 Asp is.^[42]^ If analogy between T1D Fet3p and T1HgL also holds in this respect, the T3 Glu residue is probably not involved in the pH‐dependent effect in the **RO** state of *Rv*L either

Since 2005, density functional theory (DFT) studies have been carried out on the structures of intermediates and mechanisms in MCO ORR catalysis. In the first reported study, geometries of possible key intermediates in the ORR catalysis were optimized and their relative electronic energies were compared.^[31b]^ These simulations were based on a 1.4 Å resolution crystal structure of the low‐potential MCO, CueO from *Escherichia coli*, because at the time, this one had the highest resolution of all the available MCO crystal structures.^[52]^ It was observed that the Cu−Cu distances in the crystal structure were better reproduced in the **RO**(OH; OH; –) state as compared to an **RO**(H_2_O; OH; –) state (Figure 5). The first structure was also found to be slightly more favorable in terms of its electronic energy than the latter, when the optimization was performed in a dielectric medium. In 2015, extensive QM/MM simulations of the MCO CueO by Li et al. confirmed that the **RO**(OH; OH; –) state is more stable than an **RO**(H_2_O; OH; –) state throughout the physiologically relevant pH range of the enzyme.

The combination of data obtained from experiments and simulations strongly suggest that the T2 WD‐ligand is OH^−^ and that it is remarkably stable as a function of pH. Given this pH‐independent nature of the putative T2 OH^−^ ligand, the question arises whether this group remains OH^−^ during enzyme activation or undergoes protonation to form H_2_O. The latter is tacitly suggested by Jones and Solomon in their 2015 review on the catalytic functioning of laccases, (Scheme 2, **FR** with x=2).^[21b]^ The QM/MM studies by Li et al. could not provide a definite answer, as **FR**(H_2_O; –; –) and **FR**(OH^−^; –; –) were found to be approximately in equilibrium at pH 7.^[46c]^ Nonetheless, there are indications that the mechanism presented by Jones and Solomon is indeed correct. In the first place, the earlier mentioned DFT studies by Rulíšek et al. indicated that the relative positions of the TNC Cu^I^ ions, in the **FR**(H_2_O; –; –) state matched better with the CueO crystal structure than those of the **FR**(OH; –; –) state. Although the crystal structure is in fact the **RO** state, the authors regarded this as a valid argument, since they observed the geometry of the first coordination sphere of the TNC complexes to be quite stable in going from the **RO** to the **FR** state.^[31b]^ Recently, Siegbahn also carried out DFT calculations on the **FR** state of a TNC structure optimized based on the abovementioned 1.4 Å CueO crystal structure, and found the pK_a_ of a T2 WD‐ligand to be >15. This provides a third reason for expecting protonation of the T2 OH^−^ ligand to form T2 H_2_O to actually occur under physiological conditions. Possibly, the proton involved is delivered by the T2 Asp via the H‐bonding network shown in Scheme 3.

#### 3.2.2 Thermodynamics of activation

##### 3.2.2.1 Classification of laccases based on redox properties

The thermodynamics of the activation step depend strongly on the type of laccase, as the redox potentials of laccases differ among one another. In fact, laccases can be classified according to the formal reduction potential of the T1 Cu^II^ ion, E_T1_°’. This yields high (E_T1_°’ ≈780 mV vs. NHE), middle (470 mV≤E_T1_°’ ≤710 mV vs. NHE), and low potential (E_T1_°’ ≈420 mV vs. NHE) laccases.^[53]^ It has been observed that plant laccases typically have low T1 Cu^II^ reduction potentials, whereas this redox potential is often high in their fungal counterparts. The origin of the difference in T1 Cu^II^ redox potentials has among others been sought in the differences in ligation to T1 Cu^II^ in plant and fungal laccases. However, this is still a matter of discussion that will not be further addressed here.[^11a^, ^21b^, ^54^] For most **RO** state laccases, E_T1_°’ is the only thermodynamic value reported, usually obtained using spectrochemical and/or potentiometric redox titration procedures.[^27a^, ^27c^, ^55^] Here, the discussion will be limited to laccases of which more thermodynamic data have been acquired, either by means of experiment, modeling, or by a combination of these. For tables of values of E_T1_°’ in various laccases, the interested reader is referred to Tables 4 and 5 in Shleev et al. 2005,^[11a]^ and Table 3 in Morozova et al. 2007.^[54]^

##### 3.2.2.2 Thermodynamics of activation in RvL, a low potential laccase

**Figure 6:**
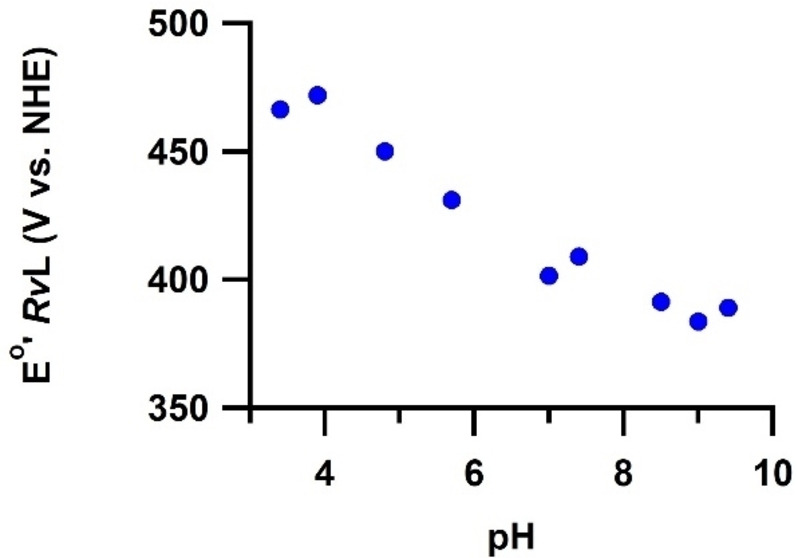
pH‐dependence of E_*Rv*L,T1_°’. Blue data: original values as reported by Nakamura, 1958.^[27g]^

*Rv*L is a typical low potential laccase and by far the enzyme for which the most redox data have been reported. Its T1 Cu^II^ formal redox potential E_*Rv*L,T1_°’ was already determined by Nakamura, via spectrochemical titration in 1958. By monitoring the 614 nm absorbance while reductively titrating the enzyme with K_3_Fe_3_(CN)_6_ E_*Rv*L,T1_°’ found to be approximately 415 mV *vs*. NHE at 298 K and pH 7.0.^[27g]^ In addition, it was observed that this formal potential showed a pH‐dependence (Figure 6). It was reported that this pH‐dependence does not arise from a proton‐coupled electron transfer. In case of proton‐coupled electron transfer, the equilibrium equation is proton‐dependent and the E^o^ can be shown by the Nernst equation to have a linear pH‐dependence. The pH‐dependence of E_*Rv*L,T1_°’ is much more irregular and was therefore suggested to arise from non‐Nernstian pH‐dependent changes in the geometry of the enzyme instead.

In studies of *Rv*L in the early 1970s, Reinhammar and Vänngård argued that Nakamura's data required re‐referencing, since the E^o^’ of the K_3_Fe_3_(CN)_6_/K_4_Fe(CN)_6_ couple is known to depend significantly on the ionic strength of the solution. Their recalculation by the ionic strength of the abovementioned potential of 415 mV yielded E_*Rv*L,T1_°’ ≈432 mV vs. NHE at pH 6.8.[^27a^, ^56^] Reinhammar and Vänngård themselves performed similar redox titrations on *Rv*L as Nakamura and expanded his work by also anaerobically titrating *Rv*L with quinoL, while monitoring by UV‐vis the 330 nm chromophore next to the 614 nm absorption band. They observed that both absorbance peaks diminished more or less simultaneously, and that the T3 coppers acted as a two‐electron acceptor. Moreover, complete silencing of the 614 nm peak occurred only after addition of four equivalents of titrant. By fitting simulated titrations of the four copper ions in *Rv*L to their experimental spectrochemical titration data, the investigators estimated E^o^’ values for the T2 Cu^II^ and the two T3 Cu^II^ ions (E_*Rv*L,T2_°’ and E_*Rv*L,*2*T3_°’, respectively) as well (Table 1, method IV).^[27a]^ Shortly after publishing these figures, Reinhammar elaborated these studies on the *Rv*L redox potential determinations by also probing them with potentiometric titration techniques in which the enzyme was titrated with ascorbate, using [Fe^II^(CN)_6_]^3−^ as a redox mediator. These results are also included in Table 1.^[55a]^ The results obtained in these potentiometric titrations did not completely converge. With different relative concentrations of *Rv*L and ferricyanide, E_*Rv*L,T2_°’ and E_*Rv*L,*2*T3_°’ shifted significantly. Based on some more experiments in which the concentrations were varied and sequence of addition of mediator and other solutes it was argued that ferricyanide probably reduces T2 Cu^II^.

**Table 1:** Redox data for various copper ions in *Rv*L All results were obtained at 298 K.

Method	pH	E_*Rv*L,T1_°’	E_*Rv*L,T2_°’	E_*Rv*L,2T3_°’	Ref.
		[mV vs. NHE]	[mV vs. NHE]	[mV vs. NHE]	
I	6.8	415	–	–	[27a,g]
I	6.8	432	–	–	[27a]
II	7.5	436	–	–	[27a]
III	7.5	390	≈390	≈390	[27a,55a]
IV	7.5	434	–	483	[55a]
V	7.5	394	≈365	434	[55a]
VI	7.0	395	365	438	[57]
VII	7.0	395	375	440	[57]
VIII	7.0	395	355	435	[57]
IX	7.0	395	365	420	[57]

I) Anaerobic spectrochemical titration of ∼0.1 mM *Rv*L with ferricyanide as redox mediator. Titrant was ferrocyanide. II) As method I, but with 0.10 mM NaF added. III) Simulated fit to results from anaerobic spectrochemical titrations of 0.1 mM *Rv*L with quinol in the presence of 0.10 mM NaF. IV) Anaerobic potentiometric titration with simultaneous monitoring of 614 and 330 nm absorbance of ∼0.1 mM *Rv*L with ascorbate and with ferricyanide as the redox mediator ([ferricyanide] : [*Rv*L]=3 : 1). Electrodes used were Pt‐Ag/AgCl with a 3 M KCl solution and a Pt‐calomel electrode with a 1 M KCl solution. V) As method IV, but with [ferricyanide] : [*Rv*L]=1 : 10. VI) Simulated fit to results from anaerobic spectrochemical titrations of *Rv*L with 1,4‐benzohydroquinone. VII) As method VI, but with xylohydroquinone as the titrant. VIII) As method VI, but with durohydroquinone as the titrant. IX) As method VI, but with [Ru(NH_3_)_6_]^2+^ as the titrant.

In 1978 Farver et al. reported additional redox data of the TNC coppers obtained from anaerobic spectrochemical redox titrations with a number of different oxidizing substrates, such as 1,4‐benzoquinone and xylohydroquinone (2,5‐dimethyl‐1,4‐benzohydroquinone).^[57]^ In their experiments, the T3 Cu^II^ did not always act as a two‐electron acceptor as in the work of Reinhammar and Vänngård. Farver et al. explained the fact that the T3 Cu^II^ ions did not invariably act as a two‐electron acceptor as a non‐equilibrium effect related to the oxidation potential of the reductant. In spite of these apparent mechanistic differences they obtained E^o^’ values for both the T1 and TNC Cu^II^ ions that were in reasonable agreement with the earlier reported values (Table 1). The validity of the data reported here is further confirmed by an earlier report by Makino and Ogura in 1970, who carried out anaerobic spectrochemical redox titrations on *Rv*L with ascorbate and H_2_O_2_ as an O_2_ analog. At pH 7.0 and 298 K they observed a redox equilibrium between the T1 copper ion acting as the electron donor and the T3 copper ions acting as a two‐electron acceptor. They determined the associated potential difference E_*Rv*L,T1_°’–E_*Rv*L,2T3_°’ to be 45 mV, which agrees well with the data obtained by Farver et al. at pH 7.0.^[48b]^

The results obtained from the redox titrations discussed above indicate that in *Rv*L, the anaerobic activation step proceeds in a more or less concerted fashion, which can be rationalized from the fact that the reduction potentials of the various Cu^II^ types in *Rv*L are quite close to one another. Several anaerobic cyclic voltammetry (CV) experiments on *Rv*L immobilized on coated Au electrodes have corroborated this concerted mechanism.[^27e^, ^58^] Results of midpoint potentials (E_1/2_) of catalytic waves in these experiments are summarized in Table 2. Invariably, only a single oxidation and reduction peak was observed with E_1/2_ values in good agreement with the results presented in Table 1. In some of these experiments a broad single quasi‐reversible redox event was found.^[58a]^ The authors ascribe the presence of a single redox event to a similar redox potential of all Cu ions. However, upon removal of the T2 Cu ion, no depletion was observed in the electrochemical behavior. It was therefore argued that direct electrochemical communication between *Rv*L and the electrode surface proceeds exclusively via the T1 Cu ion. The same single redox event was also shown in another electrochemical study on *Rv*L.^[58b]^ Here the successive scan in the cyclic voltammogram shows the depletion of the anodic current compared to the previous scan. In addition, the authors found the peak current ratio (i_pa_/i_pc_) increases from 1 to 4 with increasing scan rate. This indicates that four electrons are removed in the initial oxidative scan and that only one electron is returned in the reductive scan. Therefore the single peak could be traced back to the transfer of four electrons, i. e. a 1 : 1 anodic to cathodic peak ratio could be interpreted factually as a 4 : 4 ratio.

**Table 2:** E_1/2_ values for CV experiments on *Rv*L immobilized on coated Au electrodes.

Method	pH	Temperature	E_1/2_	Ref.
		[K]	[V *vs*. NHE]	
I	5.5	298	410	[58a]
I	7.0	298	400	[58a]
II	5.5	294–296	418	[58b]
II	7	294–296	409	[58b]
II	8.5	294–296	403	[58b]
III	6.5	298	422± 4 or 449±5	[27e]

I) Anaerobic CV with enzyme entrapped within solid, electrochemically inert tributylmethyl phosphonium chloride (TBMPC) membrane coating on Au electrode. Scan rate varied from 50–200 mV/s. II) Anaerobic CV with *Rv*L immobilized in heterogeneous orientations on an Au electrode functionalized with a 3‐mercaptopropionic acid self‐assembled monolayer (MPA SAM). Scan rate was 0.5 mV/s. III) Anaerobic CV with enzyme entrapped within solid, electrochemically inert TBMPC polystyrene cross‐linked with 1 % (w/v) divinylbenzene coating. Scan rate <100 mV/s. Note that the authors reported two different values for E_1/2_. The E_1/2_ values associated to *Rv*L and another laccase in their main text are swapped as compared to the attribution of the same values in a table.^[27e]^

Arguably, the relative values of the Cu^II^ redox potentials in *Rv*L is paradigmatic for all low potential laccases, since other MCOs with low potential T1 Cu^II^, such as ascorbate oxidase and laccase from *Melanocarpus albomyces* were featured by CV plots strongly comparable to that of *Rv*L.[^27e^, ^58a^]

##### 3.2.2.3 Thermodynamics of activation in high potential laccases

Anaerobic redox data for high potential laccases is much scarcer in comparison to the amount of data for *Rv*L, and have been reported before the first description of the **AR** state in the high potential MCOs *Ma*BOD and *Pa*L. Yet a number of experimental results obtained from several high potential laccases allows for a reconstruction of the formal redox potentials of the various Cu^II^ types in these enzymes, built on the tentative conclusion that in different high potential laccases the same types of copper ions have more or less the same redox potential. To start with, redox potentials of T1 Cu^II^ and T3 Cu^II^ ions in *Pv*L have been reported in 1972.^[55a]^ Details of these data are summarized in Table 3. Just as in *Rv*L, the two T3 Cu^II^ ions were shown to act as a two‐electron acceptor in this high potential laccase. This has been confirmed for other high potential laccases as well.[^27f^, ^47b^] Another similarity with *Rv*L is that E_*Pv*L,T1_°’ is again non‐Nernstian pH‐dependent, a feature which has also been reported for other high potential laccases.^[59]^

**Table 3:** Redox data for various copper ions in *Pv*L The results were obtained at 298 K.

Method	pH	E_*Pv*L,T1_°’	E_*Rv*L,2T3_°’	Ref.
		[mV vs. NHE]	[mV vs. NHE]	
I	5.5	785	782	[55a]
II	6.25	767	–	[60]

I) Anaerobic potentiometric titration with simultaneous monitoring of 610 and 330 nm absorbance of ∼0.1 mM *Pv*L with ascorbate and with [Fe^III^(CN)_6_]^3−^ and [Mo^V^(CN)_8_]^3−^ as redox mediators ([mediators] : [*Pv*L]=3 : 1). Electrodes used were Pt‐Ag/AgCl with a 3 M KCl solution and a Pt‐calomel electrode with a 1 M KCl solution. II) Anaerobic spectrochemical titration of *Pv*L with the [Mo^IV^(CN)_8_]^4−^/[Mo^V^(CN)_8_]^3−^ redox couple.

Next to the redox titration data shown in Table 1, several anaerobic CV experiments in which DET between high potential laccases and Au electrodes were investigated yielded some noteworthy new insights. The most important results of these experiments are summarized in Table 4. The first of these experiments concerned anaerobic CV with a capillary Au electrode in a buffered solution containing 10 mg/mL laccase from *Trametes Hirsuta* (*Th*L) without redox mediators, yielding a single catalytic peak with E_1/2_ of 405 mV *vs*. NHE. It was concluded this catalytic wave could not be assigned to DET via the T1 site, and a DET process was proposed between the T2 Cu^II^ and the electrode surface.^[55c]^ This interpretation was supported by the fact that the midpoint potential shifted to higher values after incubation of the enzyme with fluoride. Reinhammar had observed a similar effect of fluoride on E_*Rv*L,T2_°’ (Table 1, methods V and VII).[^27a^, ^55a^, ^55c^] In later CV experiments on *Th*L and *Tv*L immobilized on polymer‐coated Au electrodes two peaks were observed, with E_1/2_ values which differed by about 350 mV.^[27e]^ The lower midpoint potential E_1/2,low_ was approximately 400 mV, whereas the higher midpoint potential E_1/2,high_ had a value similar to earlier reported E_T1_°’ values of the laccases in question.^[27e]^ In light of the earlier CV results with *Th*L and the capillary Au electrode, the low potential peaks were considered as DET towards T2 Cu^II^. The high potential peaks were assigned to DET towards T1 Cu^II^. It was argued that DET from the electrode to both T1 and T2 Cu^II^ was possible due to the heterogeneous orientations in which the enzymes were immobilized on the electrode. The probability for the electrons to tunnel from the electrode surface to the enzyme's T1 Cu^II^ ions was considered to be high enough to result in an observable DET effect for that fraction of the enzymes bound to the electrode with T1 Cu^II^ in close proximity to the electrode surface. As another fraction of the enzymes is immobilized with the T2 Cu^II^ close to the electrode surface, an analogous argument can be used to explain DET from the electrode surface to the T2 Cu^II^ ions. However, there are features that point to more complex chemistry that may involve different species besides **RO** and **FR**. For example hysteresis was observed in the spectroelectrochemical titration curves *Th*L.^[55c]^

Another report worth attention is a research by Farver et al. on the kinetics of IET in anaerobic reduction of – among others – *Th*L.^[27f]^ The enzyme was sequentially reduced by means of the pulse radiolysis technique: a solution at pH 4.5 containing the enzyme, formate and redox mediators was irradiated with several consecutive pulses of electrons generated by a linear accelerator. In this process, CO_2_ radical anions were formed which reduced the enzyme exclusively via the T1 Cu^II^/Cu^I^ couple. In the meantime, the absorbance spectra at 610 nm and 330 nm were registered. These were indicative of an equilibrated IET interaction between the T1 Cu^I^ and the two T3 Cu^II^ ions. From the rate constant, a potential difference of 24 mV between both ion types was calculated. Besides, the enzyme was found to be able to take up only three electron equivalents. Farver et al. rationalized their figures by referring to the experiments as presented above in this section. In the first place, the low redox potential associated with IET from T1 Cu^I^ to the two T3 Cu^II^ ions is qualitatively comparable to the redox data for the corresponding Cu^II^ ions in *Pv*L. E_*Pv*L,T1_°’ and E_*Pv*L,*2*T3_°’ differ by 3 mV (Table 1). This could indicate that just as for the low potential laccases, E_T1_°’ and E_2T3_°’ in the high potential laccases are essentially identical as well. The larger difference between both potentials in *Th*L as compared to the difference between E_*Pv*L,T1_°’ and E_*Pv*L,*2*T3_°’ could in part be explained by the fact that the measurements on *Th*L were conducted at pH 4.5, whereas the *Pv*L redox data were obtained at pH 6.5. Since E_T1_°’ has a tendency to increase with increasing acidity, this may have resulted in a higher formal potential for IET from T1 Cu^I^ to T2 Cu^II^. Secondly, the uptake of three rather than four electron equivalents was explained by referring to the abovementioned CV experiment reporting DET between an Au electrode and the T2 copper ion in *Th*L with associated E_1/2_ of 405 mV *vs*. NHE. It was explained that there would be an insufficient thermodynamic driving force for IET from T3 Cu^I^ to T2 Cu^II^ to reduce the latter if E_*Th*L,T2_°’ were taken to be about 400 mV *vs*. NHE, and E_*Th*L,*2*T3_°’ is about 350 mV higher. However, such an incomplete electron uptake may also point to the involvement of partly reduced resting states such as **AR**.

The uptake of less than four electrons in anaerobic reduction of a high potential laccase was also noticed in early redox titrations of *Pv*L, in which the number of electron equivalents depended on the titrant used.^[55b]^ The protein was decolorized with 3.5 equivalents of [Fe^II^(CN)_6_]^4−^, and with 3.7 equivalents of [W^IV^(CN)_8_]^4−^. In both cases, no more electrons could be taken up by the enzyme. With quinol as the reducing substrate, *Pv*L took up only three electron equivalents. Monitoring the EPR spectrum of the T2 Cu^II^ showed that this copper ion was hardly reduced at all. Similarly, analysis of the T2 Cu^II^ EPR spectrum during the titration with [W^IV^(CN)_8_]^4−^ revealed approximately 30 % of the ion to have remained in the oxidized state.^[55b]^ It should be mentioned that peculiarities in the reduction of T2 Cu^II^ in high potential laccase with metal cyanide complexes were also encountered in a later redox titration of *Th*L with [Fe^II^(CN)_6_]^4−^.^[11a]^ Even after leaving a mixture of the enzyme with excess reductant for 0.5 h 18 % of the T2 Cu^II^ was still oxidized, whereas T1 Cu^II^ was readily reduced. It was also observed that the T2 Cu^II^ EPR spectrum could not be recovered upon oxygenation of the enzyme, which led to the conclusion that uncoordinated cyanide impurities present in the solutions containing the metal cyanide complex had probably complexed with the T2 Cu^II^ during the titration. Undesired side effects such as these could be responsible for the fact that in the early redox titrations electron equivalent of 3.5 and 3.7 were found rather than 3 with metal cyanides as titrants.

In generaL, the high potential laccases are probably featured by only a marginal difference between E_T1_° and E_2T3_°, which both have values of around 780 mV. E_T2_° is probably featured by an amounts to 400 mV vs. NHE. For the low potential laccases, the redox potential of each of the Cu^II^ ions is close to about 400 mV vs. NHE at pH 7.

### 3.3 PI formation

Once the enzyme is in the **FR** state, the actual ORR commences in the TNC. O_2_ associates to the TNC Cu^I^ ions and is reduced with two redox equivalents to form a peroxide moiety. Based upon MCD, EPR and SQUID experiments of T1D or T1HgLc, a diamagnetic S=0 ground state is proposed for **PI** with strong antiferromagnetic coupling between the two Cu^II^ ions.^[61]^ In some cases, comparisons are made with the paramagnetic (S=1/2 ) peroxy adduct, which is obtained upon addition of hydrogen peroxide to the **RO** of T1HgLc.[^38^, ^62^] Although, the geometric structure of the peroxy adduct is in good agreement with **PI**, the oxidation state of the TNC is different.

**Scheme 4:**
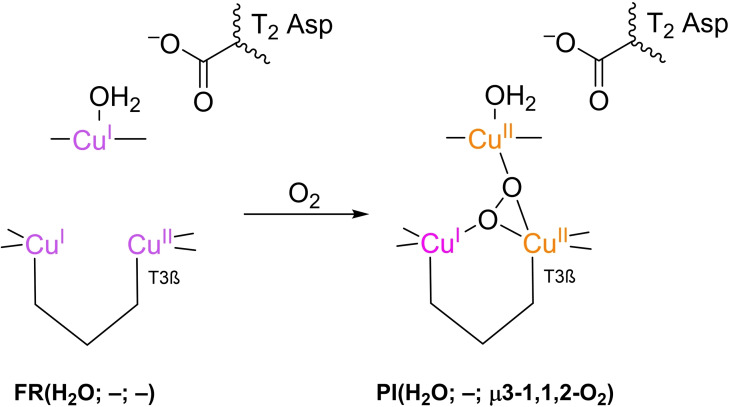
Formation of **PI** according to Solomon et al. (2010). O_2_ coordinates along the T2‐T3_β_ edge of the TNC, as the Cu^II^ ions along this edge have a lower tendency to be reduced, due to the negative charge of the carboxylate, as was predicted by *in vacuo* DFT simulations of the **PI** geometry.[^38^, ^39^] The T2 WD ligand is included as H_2_O in accordance with the way it was simulated by Yoon and Solomon in 2007.^[38]^ Note that in the simulation T2 Asp was represented by a formate group.

**PI** formation in the TNC of T1HgLc was first reported by Solomon's group in the early 1990s.^[61a]^ About a decade later, they reported this step to be independent of pH. That result was obtained from spectroscopic data from T1HgLc.^[46a]^ Around 2010, the same group showed experimentally that in T1D Fet3p, **PI** was formed under simultaneous oxidation of T2 Cu^I^ and T3_β_ Cu^I^ to Cu^II^. The asymmetry in reactivity of T3_α_ and T3_β_ was ascribed to two contributions: in the first place the T3_β_ complex has a more distorted bipyramidal geometry then T3_α_ and is therefore less stable.[^39^, ^40^] In the second place, the carboxylate moiety of the T2 Asp residue donates electron density to the T2/T3_β_ edge, effectively lowering the reduction potentials of the nearby Cu^II^ ions.^[39]^ The latter suggestion is backed by mutation studies in which reductive activity was found to be completely lost after substituting the Asp residue by alanine (Ala), whereas the rate of **PI** formation was hardly affected after substitution of Asp by Glu, which is akin to Asp in terms of its carboxylate side chain and hence in its stabilizing influence.^[31a]^ In 2007 Yoon and Solomon predicted this effect by means of DFT simulations as well.^[38]^ O_2_ association according to Solomon's group is presented in Scheme 4.

Although **PI** has never been detected in wild type (WT) laccase, there are strong indications that it is also an intermediate in the ORR cycle of WT laccase. In the first place, the TNC in WT laccase is still defined in T1D or T1HgLc.^[61a]^ Therefore, any reaction still occurring in the TNC mutant enzyme is also expectable in the WT enzyme. Secondly, the kinetics of **PI** formation in T1HgLc mutants are strongly comparable to those of **NI** formation in WT laccase.^[61a]^ The comparable kinetics were found to be maintained under various pH values.^[46a]^ Hence, from a kinetic perspective it is reasonable to regard **PI** as a precursor to **NI**. Moreover, it was reported that in T1HgLc the **PI** intermediate slowly decays to a species analogous to **NI**, and that rapid (undetectable) **PI** decay to **NI** in WT laccase is indeed to be expected.^[46b]^

**Figure 7:**
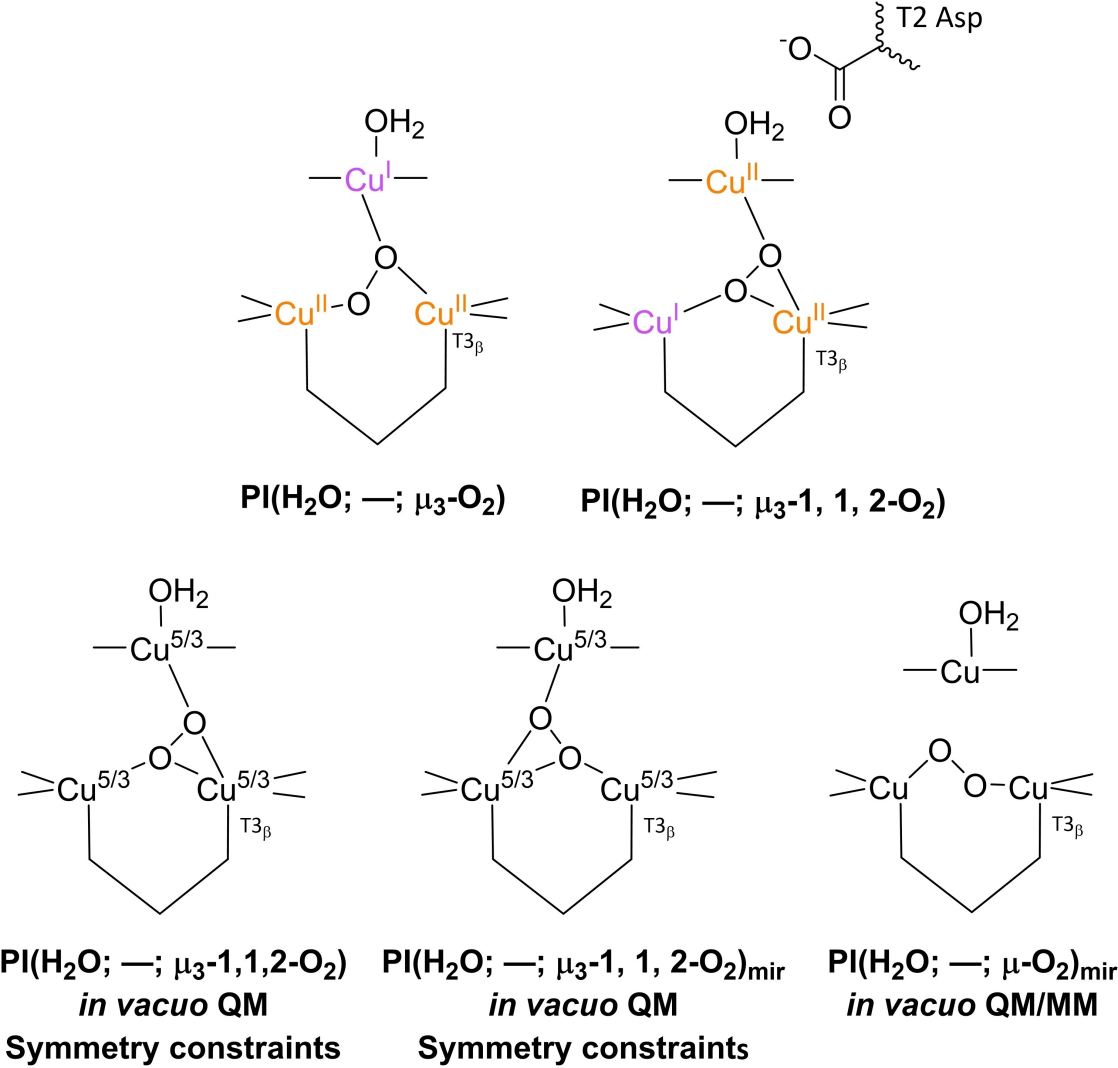
Various **PI** structures obtained in different simulation studies. Top left: **PI**(H_2_O; –; μ_3_‐O_2_) structure as proposed in simulations from 2005 and 2006.[^31b^, ^63a^] In these simulations no T2 Asp was included in the QM region. Top right: **PI**(H_2_O; –; μ_3_‐1,1,2‐O_2_) state as found by Yoon and Solomon in 2007.^[38]^ In these optimizations T2 Asp was represented by a formate group. Bottom left and center: two **PI** structures obtained by *in vacuo* pure QM DFT in 2013.^[64]^ T2 Asp was not included in the QM region. Electronic energies of both structures were the same. The Cu ions were found by ETS‐NOCV calculations to be in a mixed‐valence state. Bottom right: QM/MM optimized **PI**(H_2_O; –; μ_2_‐O_2_) structure found to be 0.0950 eV more stable than a **PI**(H_2_O; –; μ_3_‐1,1,2‐O_2_) structure optimized by the same formalism.^[64]^ For this structure no oxidation states were provided.

Pertaining to the exact structure of the TNC in the **PI** state, the literature is somewhat ambiguous. Although in none of the simulation studies in which **PI** structures were optimized, the peroxide species is protonated, its ligation to the TNC coppers and the nature of the T2 WD ligand differ from one paper to another.[^31b^, ^38^, ^46c^, ^63^] The most important structures encountered in the literature are presented in Figure 7. The first simulations in which putative structures of **PI** were optimized date back to 2005 and 2006. In both of these, the correct intermediate was considered to be **PI**(H_2_O; –; μ_3_‐O_2_) (top left structure in Figure 7).[^31b^, ^63a^]

In the 2005 article by Rulíšek et al., the motivation for considering **PI**(H_2_O; *–*; μ_3_‐O_2_) to be the correct structure was that it was the most stable of all hypothetical **PI** structures considered. A second reason was that the WD‐ligand in **FR** was expected to be H_2_O (*vide supra*), combined with the experimental result that O_2_ association is pH‐independent. The latter ruled out any protonation event via solvent water molecules or residues directly in contact with the solvent. Protonation of the peroxide ligand from a T2 WD aqua ligand was found to be energetically unfavorable. In this simulation, the spectroscopic features of the **PI**(H_2_O; –; μ_3_‐O_2_) could not be resolved, due to limitations of the DFT method applied.^[31b]^ Therefore, in 2006 multiconfigurational *ab initio* calculations by the same group were performed, leading to an electronic structure of **PI**(H_2_O; –; μ_3_‐O_2_) that was indeed in agreement with the spectroscopic characteristics of the **PI** structure obtained in experiment.^[63a]^ Note that in the 2005 and 2006 simulation studies, the QM region did not include any second‐coordination sphere residue. In the earlier mentioned DFT simulations by Yoon and Solomon, a slightly different **PI** intermediate was obtained, namely **PI**(H_2_O; –; μ_3_‐1,1,2‐O_2_), *cf*. the top right structure in Figure 7 and Scheme 4.^[38]^ In these calculations a formate functionality was included to represent the T2 Asp residue and the nature of the T2 WD‐ligand was fixed at H_2_O. Structures with OH^−^ at the T2 WD position were not considered. In 2013, Zhekova et al. also performed several DFT simulations on the structure of **PI**, primarily on small *in vacuo* models containing only the first coordination sphere of the TNC Cu ions, without including any second‐coordination sphere residue.^[64]^ They optimized **PI** structures under two different sets of optimization constraints. Under the first set of constraints all His‐ligands were kept fully frozen, and two **PI**(H_2_O; –; μ_3_‐O_2_) structures were obtained that were equally favorable in terms of their electronic energies. One of these was the same as the structure reported by Yoon and Solomon, the other one was its mirror image, accordingly referred to as **PI**(H_2_O; –; μ_3_‐O_2_)_mir_ in the present work. In the other set of *in vacuo* simulations, geometries were optimized under the same constraints as used by Yoon and Solomon in their 2007 investigations. In these constraints, only specific atoms and interatomic angles were kept fully frozen. The simulations yielded two structures, **PI**(H_2_O; –; μ_3_‐O_2_) and **PI**(H_2_O; –; μ_3_‐1,1,2‐O_2_), the former being only 0.026 eV more stable than the latter.^[64]^ These models were also subjected to extended transition state‐natural orbitals for chemical valence (ETS‐NOCV) calculations to probe their electronic structures. The outcome was that all the TNC Cu ions participate in the reduction of the coordinating O_2_ to peroxide.^[64]^ Zhekova et al. also performed QM/MM simulations, in which they included the first coordination sphere of the Cu atoms in the QM region and all other atoms within 10 Å from the TNC Cu ions in the MM region. The T2 Asp residue was included as a deprotonated residue in the MM region.^[64]^ These yielded two structures, one of which was **PI**(H_2_O; –; μ_3_‐O_2_), the other one was **PI**(H_2_O; –; μ‐O_2_), which was found to be 0.0950 eV lower in energy.

**Scheme 5:**
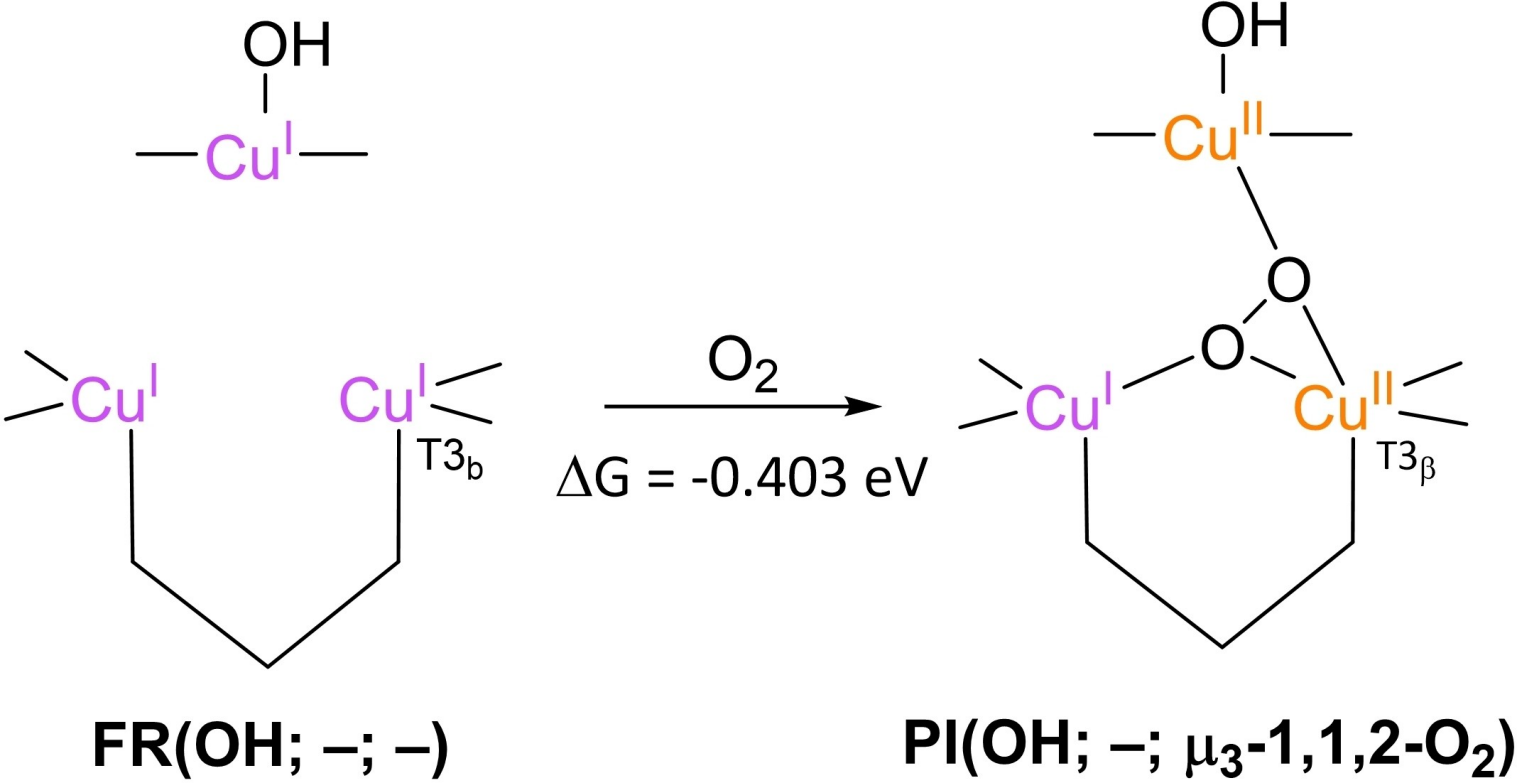
O_2_ association to **FR**(OH; –; –) to form **PI**(OH; –; μ_3_‐1,1,2‐O_2_) according to Li et al. 2015.^[46c]^ The free energy of this reaction was obtained by means of QM/MM‐PBSA calculations with B3LYP‐D3/def2‐TZVPD as the final functional/basis set combination. These QM/MM calculations were carried out in a 38 Å radius spherical system of explicit H_2_O molecules. Contributions to free energies of dissociated O_2_ were approximated by modeling unbound O_2_ in a dielectric continuum with ϵ=80, using a B3LYP functional and a def2‐TZVP basis set. The thermodynamic conditions were pH 7, 1 atm and 300 K.

All the articles discussed so far do comment on the most likely structure of **PI**, yet none of them reports calculations of the free energy ΔG of association, albeit that Zhekova et al. include some electronic energies for O_2_ association to the TNC Cu ions *(in vacuo)*. To date, only two papers have been published in which calculations of ΔG for O_2_ association to the TNC is included. The first of these was reported by Li et al. in 2015 (see Scheme 5).^[46c]^ O_2_ association to an **FR**(OH; –; –) intermediate to yield **PI**(OH; –; μ_3_‐1,1,2‐O_2_) was found to be exergonic by 0.403 eV. This result was obtained by QM/MM calculations carried out on a system based on the 1.4 Å crystal structure of the MCO CueO. The TNC QM model included imidazole rings to represent the His ligands of the first coordination sphere, and the T2 WD ligand was kept OH^−^. No second‐coordination sphere residues were included in the QM region, except an H_2_O molecule in the vicinity of the T3 Cu ions. T2 Asp and T3 Glu/Asp were only included in the MM region, as deprotonated residues.

**Scheme 6:**
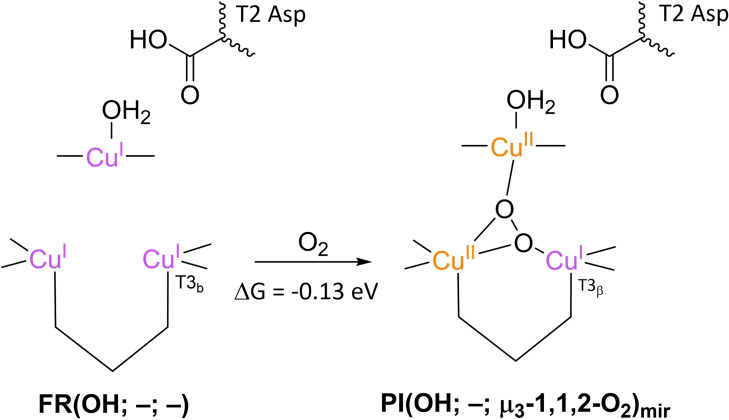
A recently proposed variant of **PI** formation: **FR**(H_2_O; –; –) reacts with O_2_ to form **PI**(H_2_O; –; μ_3_‐1,1,2‐O_2_)_mir_. Note that throughout this study, T2 Asp was modeled as a protonated residue.^[63b]^ The free energy change was calculated using DFT with a B3LYP‐D3 functional with 10 % Hartree‐Fock exact exchange, an LAV3P* basis set for the Cu ions and a cc‐pvtz(‐f) basis set for all other atoms. Solvation effects were modeled in a dielectric continuum with ϵ=4.0. The energies were calibrated to experimentally obtained data for ORR at pH 7 and a T1 Cu^II^ redox potential for the MCO in question. Oxidation states of the TNC Cu ions in the **PI** structure are complemented from Augustine et al. 2010,^[39]^ as the original paper provided no oxidation states for this intermediate. Although no thermodynamic conditions were specified, it could be deduced from the calibration method that the energies hold for thermodynamic standard conditions.

A ΔG value for O_2_ association was also calculated by Siegbahn.^[63b]^ In his work, O_2_ is proposed to associate to an **FR**(H_2_O; –; –) state to form **PI**(H_2_O; –; μ_3_‐O_2_)_mir_ with a reaction free energy of −0.13 eV (Scheme 6). This result was obtained by DFT on geometry optimized structures based again on the 1.4 Å X‐ray structure of CueO. The QM system consisted of all the first‐coordination sphere residues of the TNC Cu ions, including some crystalline H_2_O molecules and a T2 Asp residue represented by a propionic acid molecule which remained protonated throughout the entire simulation. The energies were calibrated to the experimentally obtained potential of 0.8 V *vs*. NHE for ORR at pH 7 and a T1 Cu^I^ redox potential for the CueO in question.

### 3.4 PI decay to NI

**NI** is the only intermediate that is actually observed in WT Laccase.[^46b^, ^48c^, ^65^] The structure of **NI** has been resolved by a combination of experimental and computational techniques. First of all, spectroscopic characterization of *Rv*L trapped in the **NI** state allowed the proposal of two possible **NI** structures.^[65]^ In later QM/MM simulations, the relative energies of these structures were compared as well as the extent to which their modeled electronic structure was in accordance with the spectroscopic data for **NI**.[^31b^, ^63a^] Besides, inorganic structures mimicking the TNC architecture in the then proposed **NI** structures were synthesized, of which the spectroscopic features were subsequently compared to those of the actual **NI**.[^63a^, ^66^] Based on MCD and EPR experiments a paramagnetic spin frustrated S=1/2 ground state is proposed for **NI**, wherein all three copper sites in the TNC have a +II oxidation state.[^65^, ^67^] Computational studies show a general consensus regarding this electronic state or have assumed that other spin states are not relevant.

The results of these investigations combined yielded two structures, with either a protonated or a deprotonated T2 WD ligand, *cf*. structures **NI**(H_2_O; OH; O) and **NI**(OH; OH; O) in Scheme 2. In both of these **NI** structures, all TNC copper ions are in the +II oxidation state, and the O−O bond is cleaved in a μ‐OH^−^ bridge connecting the two T3 Cu^II^ ions and a μ_3_‐oxo ligand in the center of the TNC. Later DFT simulations by Yoon et al. in 2007 confirm this finding.^[68]^

Since **NI** is the only intermediate that can be trapped in the ORR cycle of WT laccase, the exact mechanism by which reductive cleavage of the O−O bond in **PI** proceeds to yield **NI** cannot be deduced easily from experiments. Therefore, suggestions on the exact mechanism are primarily based on computational investigations.[^31b^, ^38^, ^46a^, ^46c^, ^63^, ^69^] To begin with, in 2001 Palmer et al. postulated that the step in which the actual O−O cleavage takes place in WT laccase is a process involving two electrons.^[46a]^ This postulate allowed for an explanation as to why **PI** is not detectable in WT laccase: kinetic modeling of **PI** decay to **NI** assuming two simultaneous internal electron transfer (IET) events from Cu^I^ ions to the peroxide ligand yielded a reaction rate >10^6^ times faster than slow **PI** decay to **NI** in T1HgLc. In T1HgLc, only a single electron is available for the further two‐electron reduction of the peroxide ligand, which therefore was found to be a kinetically much slower process.

**Scheme 7:**
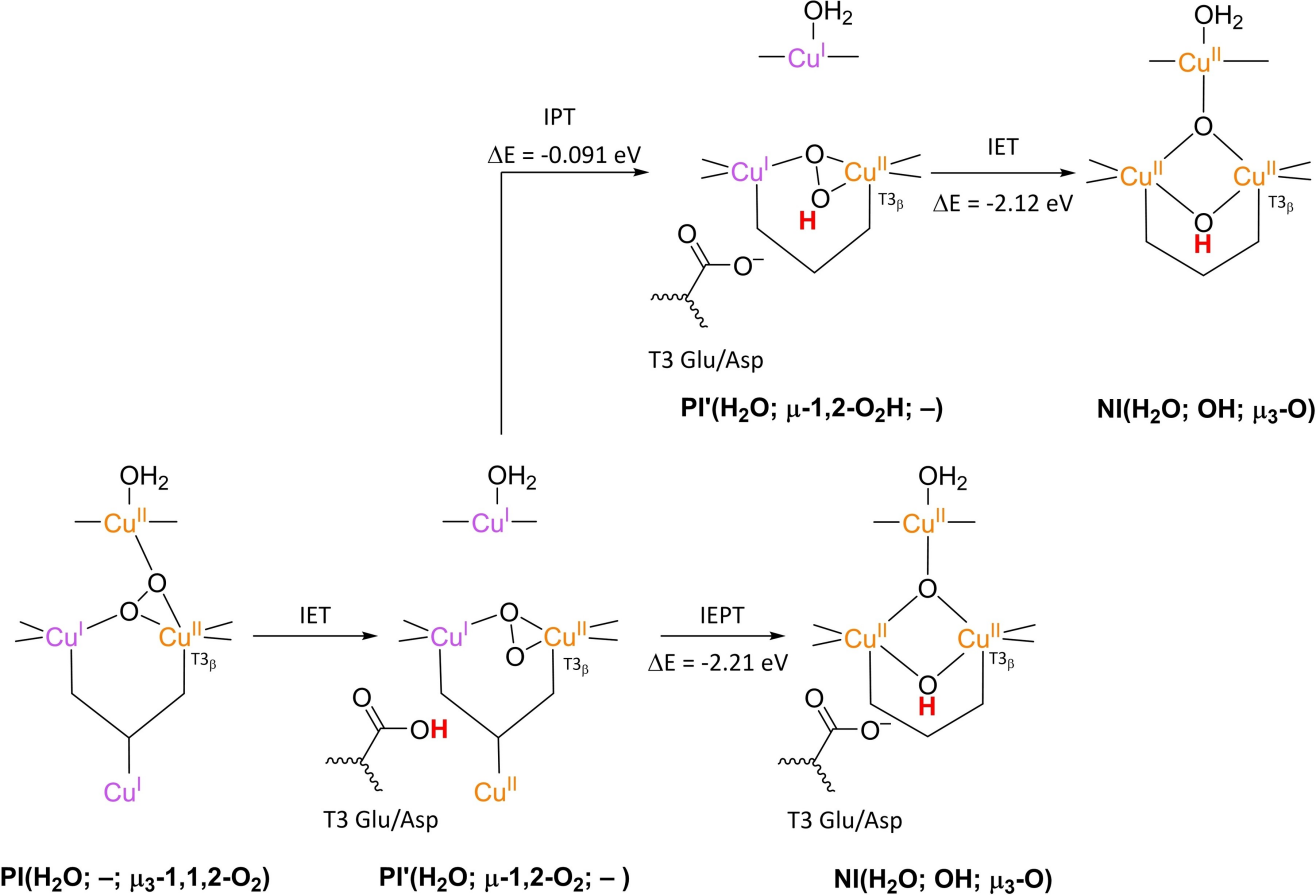
**PI** decay to **NI** in native MCOs as simulated by Yoon and Solomon in 2007.^[38]^ The first step is IET from T1Cu^I^ to peroxide ligand to yield the **PI’** intermediate, in which the O−O bond is still intact. The His‐Cis‐His bridge combined with the μ_3_‐1,1,2‐coordination of O_2_ in the TNC triangle provides an efficient electron transfer route. The subsequent cleavage is assisted by a proton donated by the T3 Glu/Asp carboxyl functionality, which was represented by a formic acid/formate residue in the actual simulations. This may occur in either a sequential IPT/IET (at low pH) or in a concerted fashion (at high pH). The IPT/IET route was proposed for acidic conditions, the IEPT route for basic conditions, in which case the proton is transferred only after passing through the transition state. In all cases, the formate representing T2 Asp was kept deprotonated and not actively involved in any of the IPT events. Changes in electronic energies were calculated with DFT on single‐points of *in vacuo* optimized TNC structures, using a B3LYP functional with a triple‐ζ 6‐311G* basis set for the Cu ions and coordinated N/O atoms; for all other atoms, a double‐ζ 6‐31G* basis set was used. Solvation effects were incorporated by modeling the system in a polarized continuum with ϵ=4.0.

As opposed to T1HgLc, WT laccase has the T1 Cu^I^ at its disposal to accomplish the fast IET of two equivalents of electrons.^[46a]^ In the first simulations of the actual mechanism of **PI** decay to **NI** in WT laccase, it was assumed that the electron from T1 Cu^I^ would be shuttled via the tripeptide link to one of the TNC copper ions still in the Cu^II^ state. In this way, the TNC has two Cu^I^ ions available to facilitate the actual two‐electron O−O cleavage to form an activated **PI** intermediate, referred to as **PI’**. Effectively, **PI** decay via **PI’** means that in the cleavage itself two electron equivalents are transferred simultaneously from two TNC Cu^I^ ions to the peroxide ligand.[^31b^, ^63a^, ^68^] In their earlier mentioned DFT simulations from 2007, Yoon and Solomon investigated two possible pathways for **PI** decay via a **PI’** transient species.^[68]^ Their results are summarized in Scheme 7. Both mechanisms share a common **PI’**(H_2_O; μ_2_‐1,2‐O_2_, –) intermediate, which is formed by the IET reduction of T2 Cu^II^ by T1 Cu^I^ via the tripeptide link and the μ_3_‐1,1,2‐coordinated peroxide ligand in the TNC. It was found in these calculations that the LUMO of T2 Cu^II^ was energetically lower than that of T3_β_ Cu^II^. In this reaction, the bond between the peroxide ligand and the T2 copper ion is cleaved. In these simulations, formic acid and/or formate residues were included in the QM model to represent the T3 Glu/Asp and T2 Asp residues, motivated by experimental observations in which these residues were found to play an important role in **PI** decay by mutagenesis studies in T1D Fet3p and T1HgLc. In the first mechanism, **PI’**(H_2_O; μ_2_‐1,2‐O_2_; –) was simulated to decay via an internal proton transfer (IPT) from the T3 Glu/Asp to the peroxide ligand to yield an additional intermediate, **PI’**(H_2_O; μ_2_‐1,2‐O_2_H; –), followed by an IET step in which the O−O bond is cleaved by two electron equivalents originating from T2 Cu^I^ and T3_α_ Cu^I^. It was argued that this was most likely the preferred pathway under acidic conditions (pH around 5), as in T1HgLc and T1D Fet3p **PI** decay was reported to be pH‐dependent.[^42^, ^46a^, ^46b^, ^61b^] For T1HgLc, the pK_a_ of the involved proton transferring group was measured to be 5.2±0.1 by Shin et al. in 1996 and to be 5.8 by Palmer et al. in 2001.[^46a^, ^46b^] For T1D Fet3p, the pK_a_ of the proton transferring group was found to be 5.0.^[61b]^

Using broken symmetry calculations, the ground state of **NI** was found to be a spin frustrated S=1/2 state, wherein all three possible states in which two different copper sites are antiferromagnetically coupled, appear to be important.^[66]^ It was reported that in particular the T3‐OH bridging ligand is important in keeping the three exchange coupling constants similar in **NI**, leading to the spin frustrated ground state.

**Figure 8:**
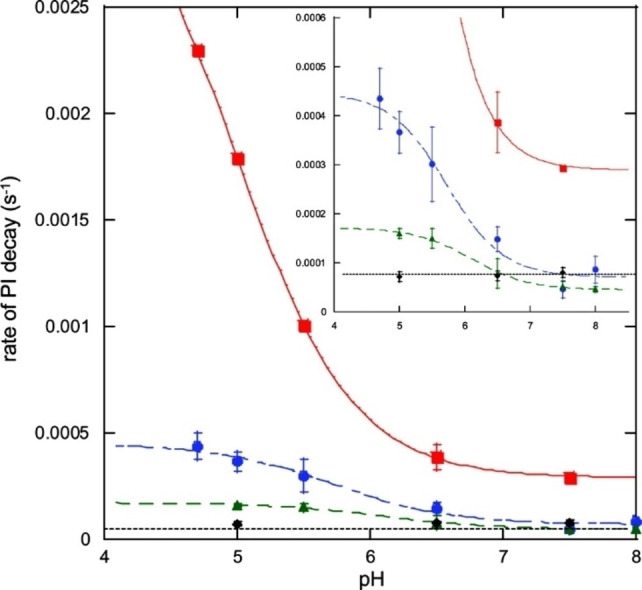
pH effects on **PI** decay rate for various mutants in the MCO T1D Fet3p. ▪: T1D Fet3p without further mutations; ♦: T1D Fet3p with T2 Asp substituted by Ala; •: T1D Fet3p with T3 Glu substituted by Asp; ▴: T1D Fet3p with T2 Asp substituted by Glu. Inset is a close‐up of the domain 0≤rate of PI decay ≤0.0005 s^−1^. Reproduced from Ref. [42]. Copyright 2007, with permission from American Chemical Society.

**Scheme 8:**
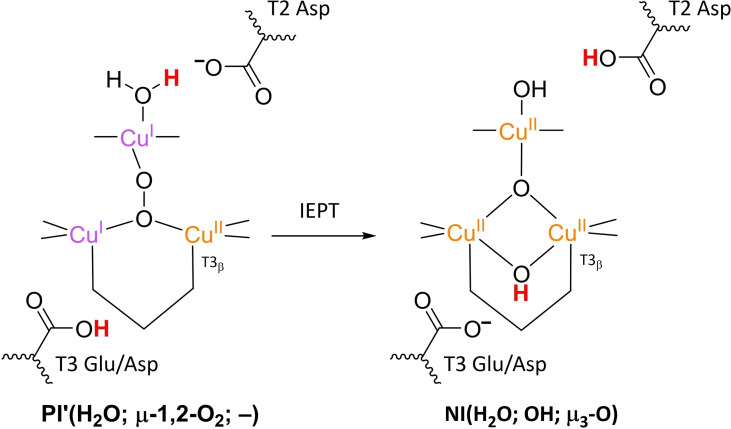
The cooperative mechanism in which both T3 Glu/Asp and T2 Asp are involved in the proton‐coupled cleavage of the O−O bond, as suggested by Augustine et al. 2007. T3 Glu/Asp donates a proton to the peroxide ligand, while T2 Asp deprotonates the T2 aqua ligand at the other side of the TNC.^[42]^

In 2007, Augustine et al. concluded from mutagenesis studies on T1D Fet3p that the group transferring the proton in **PI** decay is T3 Glu/Asp.^[42]^ The results on the pH‐dependency of the **PI** decay rate in T1D Fet3p with different mutants of this specific residue are shown in Figure 8. In T1D Fet3p, substituting T3 Glu (E487) by Ala (T1D/E487A mutant) resulted in complete loss of activity, whereas 10 % activity with respect to T1D was retained after substitution of Glu by Asp (T1D/E487D).^[42]^ In the same study, it was observed that the Asp residue in the T2 channel (D94 in Fet3p) also significantly affected the **PI** decay rate. A T1D/D94E mutant showed severe loss of activity, but to a less extent than T1D/E487D. These perceptions led the authors to suggest a cooperative mechanism, in which both the T3 Glu/Asp and the T2 Asp residue are involved in proton‐coupled O−O cleavage, (Scheme 8). The T3 Glu/Asp donates the proton required for the μ‐OH^−^ bridge, whilst the T2 Asp deprotonates the T2 H_2_O ligand during the double IET process. It was suggested that deprotonation of the T2 H_2_O ligand would also promote oxidation of T2 Cu^I^, effectively enhancing the driving force for O−O scission. A further consideration causing researchers to expect a shift in the nature of the T2 WD ligand from H_2_O to OH^−^ was that it is OH^−^ that is bound to the T2 site in the **RO** state of laccase as welL, since in both **NI** and **RO** all Cu ions are fully oxidized.^[31a]^

Although this proton donating function of T3 Glu/Asp residue was explicitly incorporated in the mechanisms simulated by Yoon and Solomon, no proton transferring activity was ascribed to T2 Asp or even investigated in these calculations and the T2 WD ligand was kept H_2_O throughout all calculations. As can be inferred from Figure 8, the pH‐dependence of the **PI** decay rate in T1D Fet3p levels off at pH ∼7.5. This was also found for T1HgLc.^[46a]^ This is consistent with a transition state (TS) in which no protons are involved, i. e. a process in which the protons are transferred only after passing through the TS.[^42^, ^46a^] Therefore, Yoon and Solomon also investigated a reactive pathway for O−O scission in which no proton was involved in the TS, whereas the proton was suggested to be still transferred in a more or less concerted fashion with the electron, referred to as internal electron and proton transfer (IEPT). In this single step, the proton is considered to be transferred only after passing through the TS. For the reaction of **PI’**(H_2_O; μ_2_‐1,2‐O_2_; –) to **NI**(H_2_O; OH; O), Yoon and Solomon calculated a change in electronic energy of −2.21 eV, *cf*. Scheme 7. This energy was split into an exergonic 0.091 EV component for IPT and a 1.12 eV contribution from the actual reductive step (IET) for the proposed mechanism under acidic conditions.

**Scheme 9:**
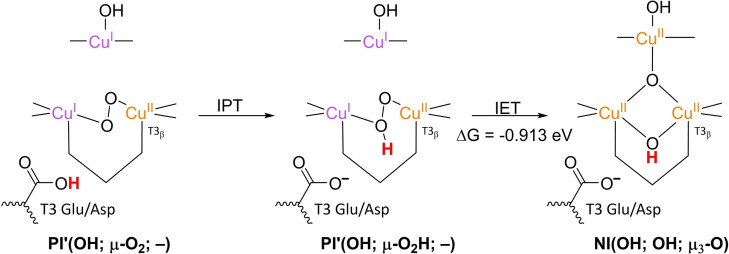
Decay of **PI’** to **NI** according to Srnec et al. 2011.^[69]^ Preliminary calculations indicated a pK_a_ increase of the peroxide ligand by 11 pH units over the course of the **PI**→**PI’** reaction. Together with the abovementioned experimentally observed pH‐dependence of **PI** decay to **NI**, this motivated the authors to propose an IPT step prior to the actual O−O bond cleavage via the IET step. The authors did not perform any explicit I(E)PT simulation, and the T2 WD ligand always remained in the same protonation state (either H_2_O or OH^−^. With OH^−^, the IET step was found to be slightly more favorable than with H_2_O). The final value of the IET electronic energy was calculated using QM/MM DFT on single‐points of geometry optimized structures with a B3LYP functional and a def2‐TZVP basis set. The enzyme was dissolved in a sphere of explicit H_2_O molecules with a radius of 38 Å.^[31b]^ To estimate the Gibbs reaction energy, thermal enthalpic and entropic contributions were estimated by means of a normal mode analysis of small *in vacuo* models of the TNC, excluding the T2 WD ligand, crystalline H_2_O and any 2^nd^ coordination sphere residues. The thermodynamic conditions of this analysis were pH 7, 298.15 K and 1 atm pressure.^[69]^

In 2011, Srnec et al. studied the double IET reaction from **PI’** to **NI** using more elaborate computational methods on QM/MM optimized structures based on the 1.4 Å CueO crystal structure.^[69]^ In the QM region, all first coordination sphere residues of the TNC coper ions were included, as well as formate groups to represent the T2 Asp and T3 Glu/Asp peripheral residues.^2^ In addition, three crystalline H_2_O molecules were included to model the H‐bond connectivity of the carboxylate residues to the T2 WD ligand and the ligand bridging the T3 coppers. ΔG values for the O−O cleavage were obtained by first calculating the associated change in electronic energy, followed by thermochemical corrections of small *in vacuo* models of the TNC in which particular elements of the QM region used for calculation of the electronic energies were omitted. Of all pathways investigated, the one found to be thermodynamically most favorable is presented in Scheme 9. Preliminary calculations of which no energetics were reported indicated that upon reduction of **PI** to **PI’**, the pK_a_ of the peroxide ligand increased by 11 pH units. This observation motivated the authors to expect the peroxide ligand to be protonated by the T3 Glu/Asp carboxyl moiety prior to the O−O cleavage reaction. In the actual O−O cleavage reaction, potential IPT events were not explicitly modeled and the T2 WD ligand was kept either H_2_O or OH^−^, nor were the energetics of the cooperative mechanism as illustrated in Scheme 8 investigated.^[69]^ It should be noted that the coordination fashion of ^−^O_2_H in **PI’**(OH; μ‐O_2_H; –) in Scheme 9 differs significantly from that encountered in **PI’**(H_2_O; μ‐1,2‐O_2_H; –), as suggested in the proton‐assisted mechanism by Yoon and Solomon, *cf*. Scheme 7.

**Scheme 10:**
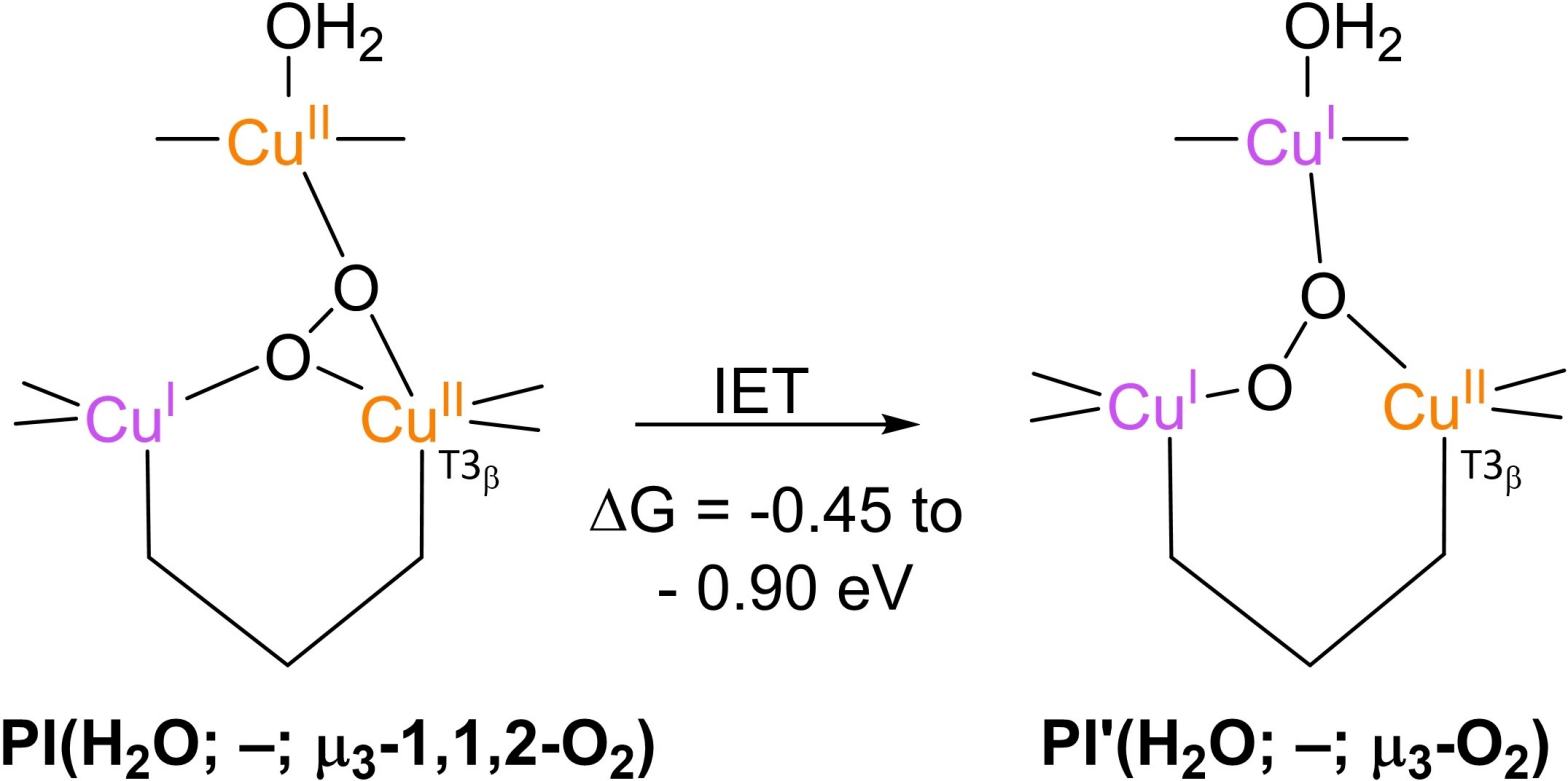
Energetics of the IET reaction converting **PI** to **PI’** according to Hu et al. 2011. The structure of **PI**(H_2_O; *–*; μ_3_‐1,1,2‐O_2_) was obtained from Yoon and Solomon 2007, and the structure of **PI’**(H_2_O; –; μ_3_‐O_2_) was based on a **PI’** structure proposed by Rulíšek et al. 2005.[^31b^, ^38^] Cu oxidation states are complemented from these sources as Hu et al. did not specify these in their paper. In all calculations a solvation shell of explicit H_2_O molecules was used with a radius of 38 Å. ΔG estimates were calculated using a Perdew‐Burke‐Ernzerhof (PBE) functional and a def2‐SVP basis set. ΔG=−0.45 eV was calculated by QTCP‐2QM methodology. ΔG=−0.90 eV was calculated using averages of QM/MM calculations of snapshots on MD simulations of the **PI** and **PI’** structures under thermodynamic standard conditions.

Next to Srnec et al. another computational study was published in 2011, in which the IET reaction of **PI** to **PI’** was simulated.^[71]^ In this article, no specific oxidation states were assigned to any of the TNC copper ions. As it was clear that the structures used were in good agreement with the work of Yoon and Solomon and Rulíšek et al., oxidation states of the Cu ions were complemented from these articles.[^31b^, ^38^] Again, the calculations were based on the 1.4 Å crystal structure of CueO. The specific structures and obtained ΔG values are presented in Scheme 10. Depending on the computational method, ΔG was found between −0.45 to −0.90 eV. The lower limit was obtained using the QM/MM thermodynamic cycle perturbation method with two QM regions (QTCP‐2QM method). The first QM region was a model of the first coordination sphere of the T1 site with the T1 Cu ion modeled either in the oxidized or in the reduced state, depending on the calculation in question. The other QM region was a model of the first coordination sphere of the TNC in which the T2 WD ligand was kept H_2_O in all simulations. The more exergonic ΔG value (−0.90 eV) was calculated using QM/MM calculations on snapshots of classical MD simulations. In these simulations, the **PI**(H_2_O; –; μ_2_‐1,1,2‐O_2_) and **PI’**(H_2_O; –; μ_3_‐O_2_) states of the enzyme were first subjected to a 10 ns MD simulation. Once every 8 ps a snapshot was taken of the enzyme, and ΔE associated with the transition from **PI** to **PI’** or from **PI’** to **PI** was calculated, depending on whether the snapshot was taken of the MD simulation of **PI** or of **PI’**, respectively. ΔE was calculated using QM/MM‐2QM methodology with mechanical embedding (ME) and the same QM regions, solvation shelL, and functional/basis set combination as in the QTCP‐2QM method. The mean of all ΔE values thus obtained was considered an to be an approximation of the free energy of the IET reaction studied, although in fact it is an average electronic energy difference. Further computational details are included in the caption of Scheme 10.

**Scheme 11:**
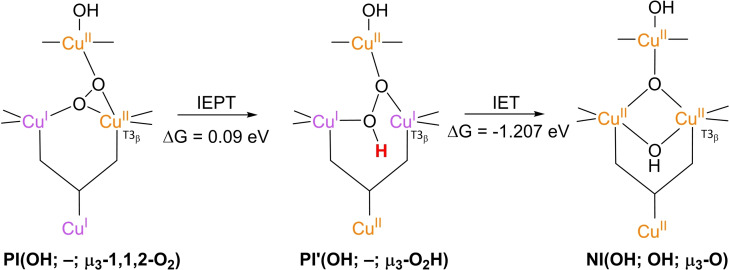
**PI** decay as proposed by Li et al. 2015.^[46c]^ In this work, both T3 Glu/Asp and T2 Asp were kept deprotonated and the T2 WD ligand was claimed to be OH throughout the catalytic cycle. The proton in the IEPT step is delivered from an Asp residue on the surface of the protein which is not involved in any H‐bonding network. This residue was chosen as proton donor/acceptor in this work for technical reasons. Note that the oxidation states of each of the Cu ions has been assigned somewhat arbitrarily in **PI**, as the authors indicated these to be hard to identify in some intermediates. For **PI’**, a T2 Cu^II^ state could be identified, rendering the T3 Cu ions in the reduced state. Free energies were obtained using QTCP‐2QM calculations with B3LYP‐D3/def2‐TZVPD as the final combination of functional/basis set. These QM/MM calculations were carried out in a 38 Å radius spherical system of explicit H_2_O molecules. Contributions of dissociated H_2_O to reaction free energies were approximated by modeling the molecule in a dielectric continuum with ϵ=80, using a B3LYP functional and a def2‐TZVP basis set.

**Scheme 12:**
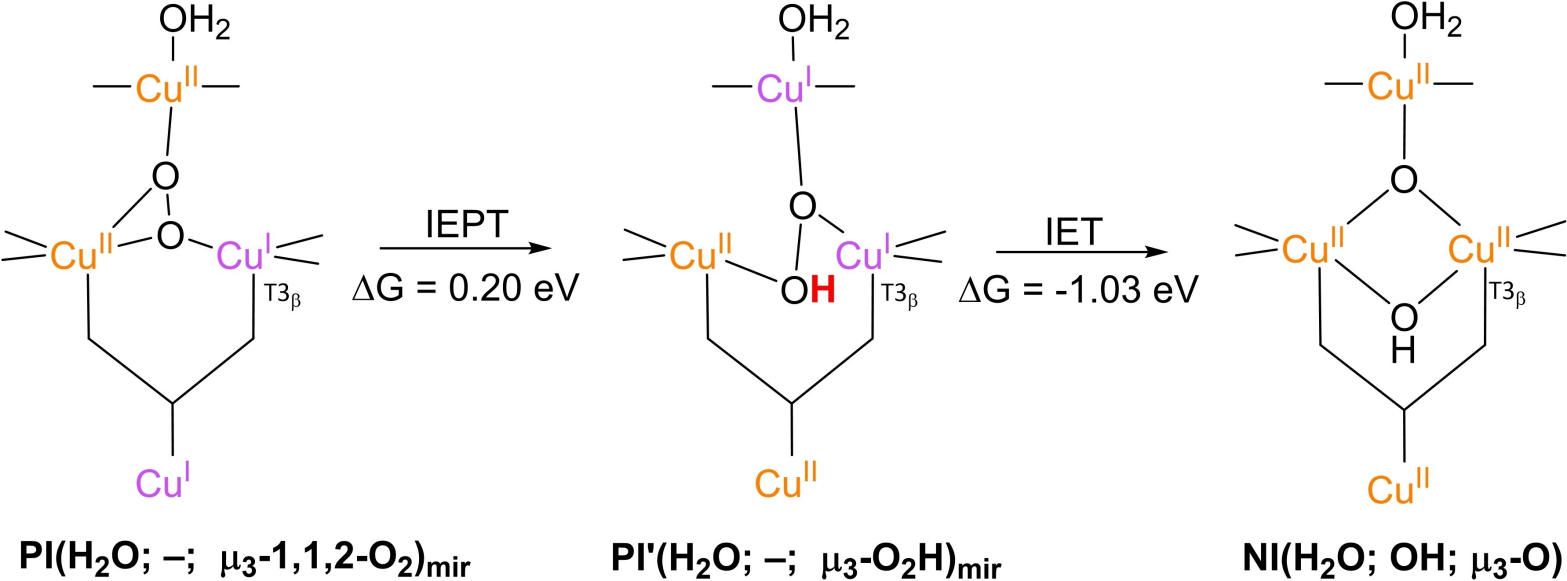
Decay of **PI**(H_2_O; –; μ_3_‐1,1,2‐O_2_)_mir_ to **NI**(H_2_O; OH; μ_3_‐O), via a **PI’**(H_2_O; *–*; μ_3_‐O_2_H)_mir_ intermediate as obtained by Siegbahn 2020.^[63b]^ In his modeL, the proton in the IEPT step was donated by solvent H_2_O at pH 7. The T2 Asp was stated explicitly to be deprotonated. T3 Glu/Asp was not considered in this simulation. The T2 WD ligand was considered to be H_2_O throughout the entire cycle. Note that in this work the oxidation state of the Cu ions in the **PI** and **PI’** structures were not specified. Free energies were calculated using DFT with a B3LYP‐D3 functional with 10 % exact exchange, an LAV3P* basis set for the Cu ions and a cc‐pvtz(‐f) basis set for all other atoms. Solvation effects were modeled in a dielectric continuum with ϵ=4.0. The energies were calibrated to experimentally obtained data for ORR at pH 7 and a T1 Cu^II^ redox potential for the MCO in question.

Li et al. and Siegbahn also incorporated **PI** decay to **NI** in their simulations.[^46c^, ^63b^] Their insights in mechanism and energetics are presented in Schemes 11 and 12, respectively. For details on the simulation procedure, see section 3.1. In Li et al. the coordination fashion of the peroxide ligand is identical to that in the simulations by Yoon and Solomon 2007, *cf*. the **PI** structures in Schemes 7 and 10.[^38^, ^46c^] In contrast to the mechanism proposed by Yoon and Solomon, Li et al. suggested the T2 WD ligand to be OH^−^ throughout the entire catalytic cycle. **PI**(OH; –; μ_3_‐1,1,2‐O_2_) was proposed to decay to **NI**(OH; OH; O) via an IEPT step to form **PI’**(OH; –; μ_3_‐O_2_H), followed by IET from the copper ions to the ^−^O_2_H ligand. The reaction energies were +0.09 eV and −1.207 eV respectively. Li et al. 2015 did not always assign formal oxidation states to their structures. These have been complemented from other sources or have been omitted in case of ambiguity.[^31b^, ^38^]

The mechanism obtained by Siegbahn contained some new suggestions (Scheme 12).^[63b]^ The most striking difference with previous mechanisms is the **PI**(H_2_O; –; μ_3_‐1,1,2‐O_2_)_mir_ structure, which is similar to that optimized by Zhekova et al. 2013.[^63b^, ^64^] As in the mechanism proposed by Li et al. **PI** is converted to **PI’** via a slightly uphill IEPT reaction, this time ΔG amounted to +0.20 eV. The actual O−O scission is a double IET event as in all other simulations, which is downhill by 1.03 eV.^[63b]^

### 3.5 NI re‐reduction to FR

Once the **NI** is formed, the sequel of the reaction depends on whether more O_2_ is present for reduction or not. If the enzyme and its environment are depleted in O_2_, the enzyme decays to the **RO** state, as depicted in Scheme 2. Alternatively the **NI** state decays to the partly reduced **AR** state by lack of O_2_ in case of the high potential MCOs *Ma*BOD and *Pa*L.[^47a^, ^47b^] If O_2_ is still present, all four Cu^II^ ions of **RO** are rapidly re‐reduced in a process involving four electrons and four protons. The electrons are provided via four sequential substrate oxidations.[^45^, ^68^] Water is probably excreted via the hydrophobic channels.[^32a^, ^34c^, ^34d^, ^34e^, ^34f^] The decay rate of **NI** to **RO** is over a thousand times lower than the rate of catalytic turnover as observed in substrate oxidation by *Rv*L or *Tv*L.[^30c^, ^45^] Therefore, **RO** cannot be part of the catalytic ORR cycle. In part, this can be explained by the fact that **RO** lacks the strongly basic μ_3_‐oxo bridge, which was found to be pivotal in the proton coupled re‐reduction of the **NI**. Together with the μ‐OH^−^ bridge, it facilitates rapid internal electron transfer (IET) steps, as these bridging ligands allow delocalization of electrons over all three TNC copper ions. Next to that, their high proton affinities contribute to the thermodynamic driving force during various stages of the re‐reduction step.[^37^, ^45^, ^68^]

**Scheme 13:**
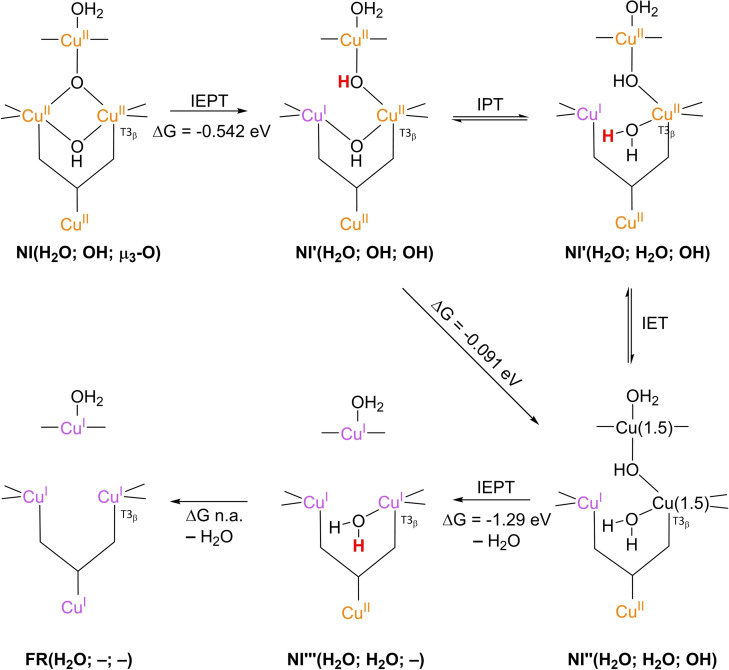
Simulated mechanism for regeneration of the **NI** state by Heppner et al.^[37]^ The last reductive step was not included in the simulation. Free energies were obtained by means of DFT calculations on single points of a geometry optimized QM system including the TNC first coordination sphere, two crystalline H_2_O molecules of which one was the T2 WD ligand, the other H‐bonded to the μ‐OH^−^ ligand (not shown) and a formate moiety to represent the T2 Asp residue. T3 Glu/Asp was generally not explicitly included, although this residue was mentioned as the proton‐shuttling group. Initial energy calculations were performed with a B3LYP functionaL, a TZVP basis set for the Cu ions and coordinated N/O atoms and a TZV basis set for all other atoms, with QM system embedded in a dielectric continuum with ϵ=4.00; a proton solvation energy of 11.30 eV was used to model PT steps. As the initial energies thus computed turned out be invariably much too exergonic, they were calibrated by a method based on the experimentally obtained free energy of reduction of the **RO** state of *Rv*L.[^37^, ^55a^] In this so‐called ‘resting calibrated’ method, the free energy of reduction of the MCO model in the **RO** state was first calculated by the initial computational method, to yield the overestimated ΔG value. Next, this value was compared to the experimentally obtained free energy of **RO** reduction to provide a quantitative indication of the overestimations made in the initial calculations. The absolute value of this indication was subsequently added to the initially calculated free energies of all the other catalytic steps simulated.

Experimental studies aimed at elucidation of the re‐reduction pathway are very limited, since the kinetics of this part of the ORR cycle are extremely fast and beyond the rate determining step. Upon reacting *Rv*L with 5 equivalents of hydroquinone and a single equivalent of O_2_ Heppner et al. perceived that the T1 copper remained practically oxidized, whilst the TNC Cu^II^ ions were sequentially reduced. As hydroquinone reduces T1 Cu^II^, it was concluded that the IET rate k_IET_ from T1 Cu^I^ to TNC Cu^II^ ions with the associated re‐oxidation of the T1 Cu^I^ must be rapid (k_IET_ >700 s^−1^).^[45]^ In the same report, it was established that the first step of the re‐reduction is a proton‐coupled ET (PCET) event in which the T3_α_ Cu^II^ is reduced and the basic μ_3_‐oxo bridge is protonated; proton and electron are transferred simultaneously (IEPT) (Scheme 13, first step from **NI**(H_2_O; OH; μ_3_‐O) to **NI’**(H_2_O; OH; OH)). This occurs hand in hand with loss of the bond between the T3_α_ copper ion and the μ_3_‐OH^−^ ligand. In 2014, the proceedings of the re‐reduction were further examined by DFT simulations, the results of which are depicted in Scheme 13, including ΔG values.^[37]^ These revealed that the next step is rapid PT to the μ‐OH^−^ bridge between the T3 coppers to yield **NI’**(H_2_O; H_2_O; OH), followed by ET in which a mixed‐valent T2/T3_β_ copper pair is formed (**NI’’**(H_2_O; H_2_O; OH)). The third step is again a PCET in which this copper pair is reduced to two Cu^I^ ions under protonation of the μ‐OH^−^ ligand connecting them (**NI’’’**(H_2_O; H_2_O; –). It was found that excretion from the TNC of the water produced in this process contributed significantly to the energy gain achieved in turnover.^[37]^ The QM system was based on the 1.9 Å crystal structure of *Tv*L. It was defined by the TNC's first coordination sphere, two crystalline H_2_O molecules of which one was the T2 WD ligand, the other H‐bonded to the μ‐OH^−^ ligand (not shown), and a formate moiety to represent the T2 Asp residue. T3 Glu/Asp was generally not explicitly included, although this residue was mentioned as the proton‐shuttling group.^[37]^ Further technical details of the energy calculations and how they were calibrated are included in the caption of Scheme 13.

**Scheme 14:**
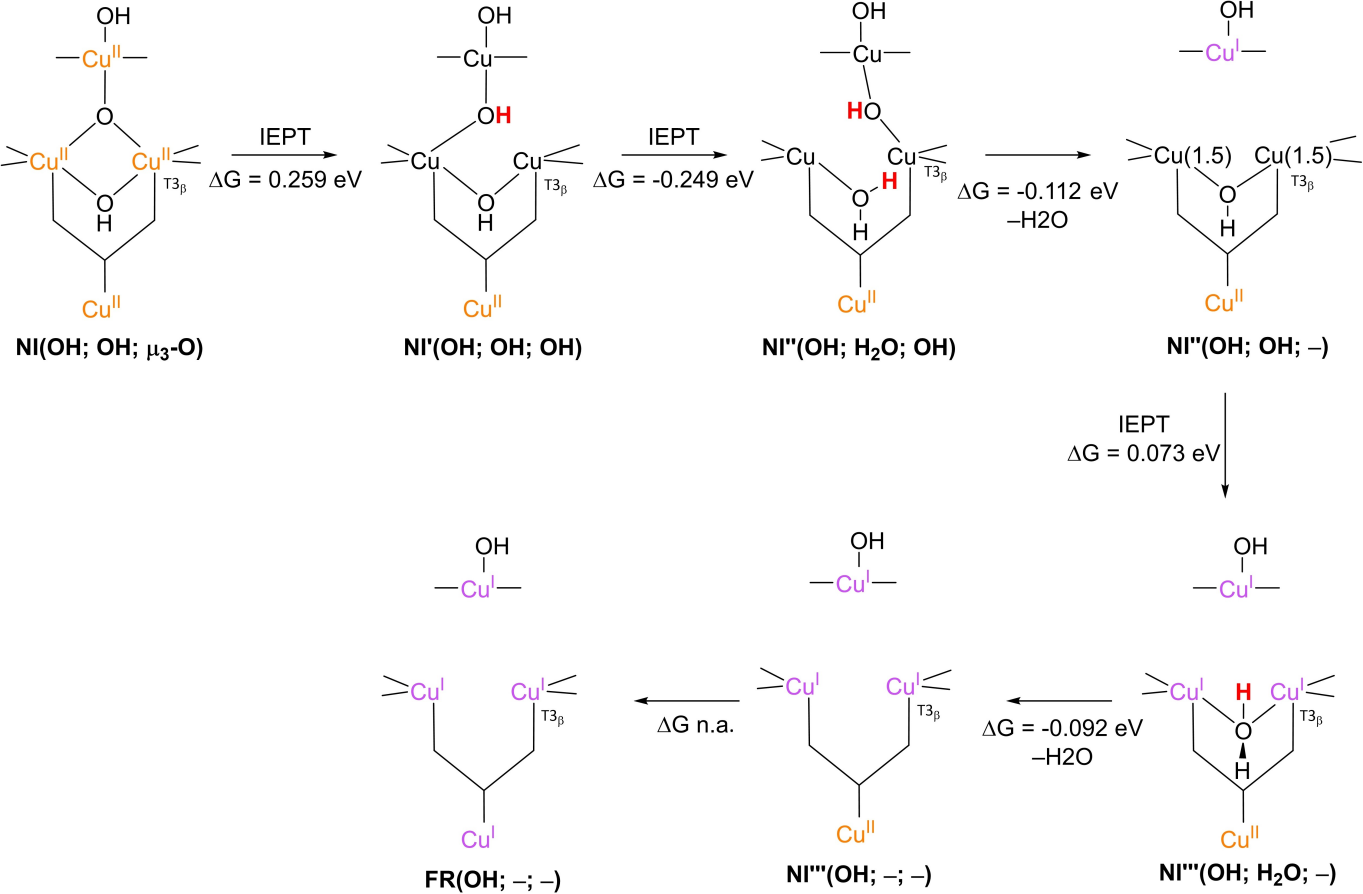
The second suggested mechanism for regeneration of **FR** from **NI**.^[46c]^ The last reductive step was not included in the simulation. Free energies were obtained using QTCP‐2QM calculations with B3LYP‐D3/def2‐TZVPD as the final combination of functional/basis set. These QM/MM calculations were carried out in a 38 Å radius spherical system of explicit H_2_O molecules. Contributions of dissociated H_2_O to reaction free energies were approximated by modeling the molecule in a dielectric continuum with ϵ=80, using a B3LYP functional and a def2‐TZVP basis set.

The re‐reduction pathways suggested by Li et al. 2015 differs significantly from the one obtained by Heppner et al. in 2014, both in terms of the mechanism, as well as the free energies associated to individual steps, (Scheme 14).^[46c]^ Nonetheless, there are also similarities. All ET events are in some way proton‐coupled, and in both mechanisms mixed valent states are involved. Moreover, Li et al. 2015 also calculated IET reactions from T1 Cu^I^ to formally oxidized Cu ions in the TNC by explicitly including a model of the T1 first coordination sphere, with the T1 copper simulated as either Cu^I^ or as Cu^II^, thus allowing for a more genuine simulation of IET. Regarding the differences, Li et al. 2015 first of all consider the T2 WD ligand to be OH^−^, whereas the same ligand is kept H_2_O by Heppner et al. 2014. Secondly, the intermediates generated in first IEPT step are mirror images of one another, at least regarding the ligation of the central OH^−^ ligand. Something similar is true for the aqua ligands in the **NI’’** structures. In the structures NI’(H_2_O; H_2_O; OH) and **NI’’**(H_2_O; H_2_O; OH) by Heppner et al. H_2_O and OH^−^ coordinate to the T3_β_ ion, whereas in the analogous structures by Li et al. (**NI’**(OH; OH; OH) and **NI’’**(OH; H_2_O; OH) H_2_O and OH^−^ coordinate to the T3_α_ ion. Another difference is that in the mechanism proposed by Heppner et al. the H_2_O products are excreted quite late during the re‐reduction process. Contrary to that, Li et al. expect H_2_O to be produced and excreted quite early on in the chain of reactions. Furthermore, the ligation of the H_2_O in between the two T3 Cu^I^ ions is different in **NI’’’**(H_2_O; H_2_O; –) and in **NI’’’**(OH; μ_2_‐H_2_O; –), *cf*. Schemes 13 and 14, respectively.

**Scheme 15:**
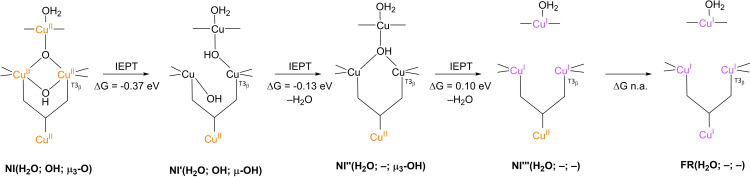
A third re‐reduction mechanism recently proposed by Siegbahn.^[63b]^ The last reductive step was not included in the simulation. Free energies were calculated using DFT with a B3LYP‐D3 functional with 10 % HF exchange, an LAV3P* basis set for the Cu ions and a cc‐pvtz(‐f) basis set for all other atoms. Solvation effects were modeled in a dielectric continuum with ϵ=4.0. The energies were calibrated to experimentally obtained data for ORR at pH 7 and a formal redox potential of T1 Cu^II^ in CueO.

The mechanism and energetics of re‐reduction as calculated by Siegbahn are presented in Scheme 15.^[63b]^ Siegbahn's mechanism is much more concise than the other two re‐reduction mechanisms, as H_2_O is simulated to excrete directly in several IEPT steps without modeling any actual dissociation process. Besides, the intermediates presented by Siegbahn differ from those obtained by either Heppner et al. or Li et al. The OH^−^ ligand in **NI’**(H_2_O; OH; μ‐OH) is coordinated to the T3_α_ copper only, rather than both T3 coppers as in the **NI’** structures obtained in the other two simulations. A μ_3_‐coordinated central OH^−^ ligand as present in **NI’’**(H_2_O; –; μ_3_‐OH) is not observed elsewhere either.

None of the articles discussed so far explicitly calculated a free energy change for the final reduction of T1 Cu^II^ to bring the re‐reduction to completion. However, in 2016 Götze and Bühl published ionization energies of T1 Cu^II^ on an **NI’’’**(OH; –; –) QM/MM model.^[72]^ Depending on the protonation state of the enzyme, they obtained a redox potential of an ionization energy of 0.880 V *vs*. NHE (low pH) and −0.510 V *vs*. NHE (high pH) at 300 K and 1 atm pressure. The specific pH of each calculation was left undefined. These numbers were calculated by taking 10 snapshots from a 1 ns MD simulation of a laccase model based on the 1.9 Å crystal structure of *Tv*L, with a 35 Å radius sphere of explicit H_2_O molecules centered on the T1 site of the enzyme. For each snapshot, a QM/MM optimization of the T1 Cu complex was carried out, with the T1 copper ion simulated either as Cu^I^ or as Cu^II^ in all snapshots, or starting the simulation with T1 Cu^II^ and gradually converting to a T1 Cu^I^ state in the subsequent snapshots. The redox potentials were calculated with an M06 functionaL, an SDD basis set for the copper ion and a 6‐311++G** basis set for all other atoms in the QM region.

## 4 Discussion and recommendations

### 4.1 Current progress

This review aims to identify the challenges complicating the elucidation of an unambiguous FES vital to understanding the low overpotentials at which laccases have been shown to catalyze the ORR. Many crucial building blocks required for the laccase‐catalyzed ORR FES are already present and have been reviewed in the previous sections. To start with, the TNC structure of the resting enzyme is well defined for a large number of MCOs and its general mechanism is well‐established, and based on an extensive body of spectrochemical and crystallographic evidence.^[21b]^ Secondly, the key steps of the ORR reactive cycle as well as the cardinal structural features of key intermediates have reached a status of consensus. For example, the peroxide ligand in the **PI** is known to be coordinated to all of the TNC coppers, and the **NI** is featured by a central μ_3_‐oxo ligand and μ‐OH^−^ bridging the T3 Cu ions.[^31b^, ^38^, ^63a^, ^64^, ^65^, ^66^, ^68^] For all key intermediates, the structure could be inferred from a combination of crystallographic, spectrochemical and computational data. Moreover, various steps in MCO catalyzed ORR have been analyzed in computational studies, especially during the last fifteen years.[^21b^, ^31b^, ^37^, ^38^, ^46c^, ^63^, ^69^, ^71^, ^72^] Notwithstanding this experimental and computational progress, construction of a clear and complete FES for laccase catalyzed ORR is faced with persistent challenges. On the one hand, these are fundamental in nature. On the other hand, the mechanistic and thermodynamic unclarities are related to mutual differences in the systematics of computational investigations undertaken thus far. Both types of challenges are discussed respectively in the following.

### 4.2 Fundamental challenges

#### 4.2.1 Kinetic challenges

The tradeoff between fast kinetics and fully fledged mechanistic elucidation by means of experiment is one of the most straightforward challenges for determining the FES. Trapping and characterizing intermediates becomes increasingly more peculiar if the reactive chain is followed with a higher rate. In the case of laccase‐catalyzed ORR, only **RO**, **FR** and **NI** are detectable in the native enzyme.[^46b^, ^48c^, ^65^] **PI** is not detectable in WT laccase, but could be observed in MCOs with mutated T1 sites.[^39^, ^61a^] **AR** was only detected in case of PaL.^[47b]^ Most of the elementary steps from one of these intermediates to another and the associated energetics cannot be probed by spectrochemical or by voltammetric techniques. Consequently, the only remaining tool for finding the mechanistic details of elementary steps is simulation, with only a limited amount of experimental data for validation at hand.

#### 4.2.2 Electrochemical challenges

**Figure 9:**
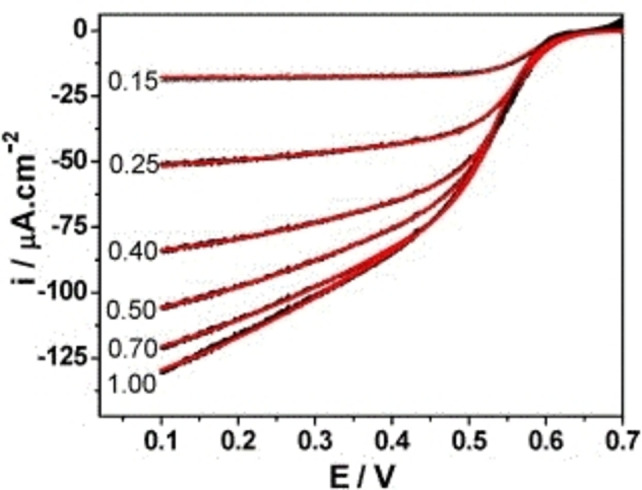
Typical plateau currents observed in voltammetric studies of the ORR catalyzed by laccase via DET from an electrode to the T1 Cu^II^/Cu^I^ redox couple. Polarization curves are displayed for ORR catalyzed by laccase immobilized on a graphite electrode under different partial pressures of O_2_. Numbers prior to each curve represent mole fractions of O_2_ in a mixture containing N_2_ and O_2._ The potential is given with respect to an Ag/AgCl reference electrode with 3 M KCl. Reproduced from Ref. [14]. Copyright 2016, with permission from Elsevier.

Concerning the limited body of experimental data, fundamental challenges are present particularly in the electrochemical field. For none of the ET events in any of the catalytically relevant steps standard potentials can be determined by means of redox titrations, as this would require the elementary steps to be possible in isolation, which is disabled by the cycle's rapid kinetics. Studies that involve voltammetric techniques were not conclusive either. When catalyzing the ORR using laccase immobilized on an electrode the observed maximum current densities are often limited by the diffusion of reactants to the TNC rather than by limitations in the enzyme. Consequently, voltammograms of laccase catalyzed ORR are not featured by catalytic waves which can be associated to specific elementary steps. Instead, the voltammograms are characterized by plateau currents which do not provide further mechanistic or thermodynamic insights.[^12^, ^14^, ^27e^, ^58b^, ^73^] An example of plateau currents is provided in Figure 9, which shows cathodic polarization curves for a graphite electrode with immobilized laccase under various partial pressures of O_2_. All curves level off with increasingly negative potential. There were polarization curves were performed at conditions where the rate is limited by the catalyst concentrations, kinetic parameters could however be derived, upon extensive data analysis employing numerical methods and modeling.[^47c^, ^74^] Any determination of thermodynamic parameters by e. g. differential pulse techniques that are of use to extract a potential energy surface has not been reported thus far. In fact, redox data have only been obtained for the slow, catalytically less relevant steps, such as the anaerobic reduction of **RO** to **FR**, and even for this step knowledge is scarce. For many laccases, only formal potentials of the T1 Cu^II^/Cu^I^ couple have been published.[^27a^, ^27b^, ^55^] Only for a handful of laccases TNC redox potentials or potentials associated with IET from the T1 Cu to the TNC have been deduced from redox titrations, DET experiments and pulse radiolysis.[^27a^, ^27e^, ^27f^, ^48b^, ^55a^, ^55c^, ^57^]

The drawbacks in determining redox potentials of the TNC's Cu^II^/Cu^I^ couples in **RO** mainly arise from the fact that directly reducing the TNC Cu^II^ ions is extremely difficult, as the TNC is embedded in the interior of the enzyme. In redox titrations, reducing substrates usually react exclusively at the T1 site. Besides, the spectroscopic features of the T2 Cu^II^/Cu^I^ are unsuitable for most spectroelectrochemical titrations, although in theory titrations using EPR should be feasible. Actually, formal potentials of this T2 couple have only been reported for *Rv*L and were determined indirectly by using a combination of spectroelectrochemical titration data and computational modeling.[^27a^, ^55a^] Direct communication of an electrode surface with the TNC copper ions is difficult and if at all possible, it seems to occur via the T2 copper only.[^27e^, ^55c^] In low potential laccases such as *Rv*L, the formal potentials of the different Cu^II^/Cu^I^ couples are so close to one another that individual redox peaks, if any, are indistinguishable in voltammetric experiments.[^27e^, ^58^] Only for few high potential laccases DET from the electrode to the T2 Cu^II^/Cu^I^ has been observed next to DET communication with the T1 Cu^II^/Cu^I^ couple.[^27e^, ^55c^] Although the distance from the T3 site to the outer periphery of the enzyme is roughly 14 Å,^[32a]^ in practice the electron tunneling probability from the electrode to the T3 Cu ions is apparently very low. However, these studies are difficult to interpret for various reasons, and a more thorough understanding of the potential involvement of partly reduced resting states in high potential laccases may be crucial to fully understand the complexity of these redox reactions.

Pertaining to the reduction potentials that have been published, these often lack general applicability, because they are formal/apparent potentials, which are no true standard potentials. True standard potentials are invariant properties of a redox couple. These can be used theoretically to calculate the equilibrium potential of the redox couple under any conditions using the Nernst equation, provided that the activities of reductant and oxidant are known. This condition was not met in the redox titrations of laccase, in which only the concentrations were directly measurable. Therefore, in the Nernst equation, the actual concentrations are used rather than the activities. From the pH‐dependence of E_T1_° as reported by Nakamura for *Rv*L, it becomes apparent that the activity coefficients of the Cu ions in laccases are pH‐dependent functions in a non‐Nernstian fashion.^[27g]^ As such, the fact that the formal potential is not stable under varying thermodynamic conditions is not surprising since the geometry and protonation state of enzymes and hence their activities are strongly pH‐dependent. In 2016, Götze and Bühl observed such a non‐Nernstian pH‐dependence qualitatively in calculations of absolute redox potentials of the T1 site in *Tv*L under different protonation states of the Glu and Asp residues in the enzyme.^[72]^ They found that the protonation state of especially residues within 20 Å from the T1 Cu ion strongly affect this ion's redox potential. There are no quantitative expressions relating the activity of laccases in specific oxidation states to the thermodynamic conditions. Consequently, the actual standard potential is not known, and the formal potentials that are available can strictly be used only for conditions under which they were determined. Probably the TNC Cu^II^/Cu^I^ redox potentials have non‐Nernstian pH‐dependencies as well. This could be verified by repeating the experiments by Reinhammar and Vänngård over the pH range of physiological relevance to *Rv*L. Such a redetermination of redox potentials of the Cu^II^/Cu^I^ redox couples at various pH‐values could also shed light on the pH‐dependence of the reduction of T3 copper ions. If this reaction is indeed proton‐coupled within the physiologically relevant pH‐range of the enzyme, a pH‐dependence is expected for this pH‐domain. Besides, it would be useful to map the variability of the formal potentials as a function of other factors that could influence the formal potentiaL, such as the ionic strength of the electrolyte solution.

In spite of all these drawbacks, the published redox data allow for a rough estimation of redox potentials in the **RO** to **FR** reaction in low and high potential laccases. For the low potential laccases, all three Cu^II^/Cu^I^ couples are characterized by apparent redox potentials of ∼400 mV *vs*. NHE at pH 7. For the high potential laccases, the T1 and T3 Cu^II^/Cu^I^ couples all have similar potentials of about 780 mV *vs*. NHE at pH 7. At pH 7, the T2 Cu^II^/Cu^I^ couple has a formal potential of ∼400 mV *vs*. NHE. These data are probably not of much use for redox events further down the catalytic cycle, however: computational evidence shows that the redox potential of the T1 Cu^II^/Cu^I^ couple is strongly dependent on the net charge of the TNC Cu ions and their direct ligands. For various catalytically relevant intermediates in which the TNC had a net charge ranging from 2 to 3 e^+^, the T1 redox potential varied significantly by 180 mV.^[46c]^ For higher TNC oxidation states, this variation was even larger. The documented redox data only hold for the **RO** to **FR** step and are therefore unsuitable to make predictions of the redox potentials of ET reactions in the ORR catalytic cycle. In short, this means that obtaining the electrochemical details of the catalytically relevant steps faces the same challenge as the elucidation of the mechanistic details. They can only be estimated based on computational research. Although calculating standard potentials is extremely demanding, various methods exist that give reasonable results, such as those adopted by Hu et al. 2011 or Li et al. 2015.[^46c^, ^71^] In such future studies it would be helpful to validate the quality of the method used for calculating redox potentials by simulating the **RO** to **FR** reaction and comparing the results with the experimental figures.

#### 4.2.3 Challenges related to TNC structure and function

The high degree of canonicity in the active site in many of the MCOs has led to the fundamental assumption that all MCO's catalyze ORR in the same manner, as the same structural features are expected to behave according to similar mechanisms. Regardless of the validity of this assumption, there seem to be some contradictory claims in the literature concerning the nature of the T2 WD ligand and the role of the T2 Asp. The mutant studies by Solomon's group led to the conclusion that T2 Asp is probably a proton‐transferring moiety, which plays an important role in protonation and deprotonation of the T2 WD ligand in specific catalytic steps such as **PI** decay to **NI**, (Schemes 3 and 8).[^31a^, ^41^, ^42^]

**Figure 10:**
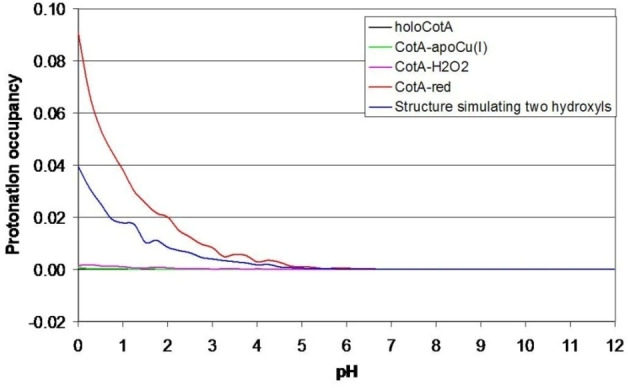
Proton‐inactivity of T2 Asp as observed in simulated pH titrations of this residue in various CotA structures. The red curve belongs to CotA in the **FR** state; the curve in magenta resembles the **PI** structure. Reproduced from Ref. [34e]. Copyright 2010, with permission from Springer nature.

Contrary to these proposals, in simulated pH titrations of T3 Glu and T2 Asp in CotA laccase Bento et al. found that T2 Asp remains deprotonated throughout the entire physiological pH range in both the **FR** and **PI** state of the enzyme (Figure 10).^[34e]^ In these simulations, the T2 ligand was kept H_2_O, although OH^−^ was modeled loosely by adding an extra charge to the aqua ligand. No PT close to pH 7 was observed either, whereas T3 Glu shuttles protons in this pH‐range. Consequently, the authors interpreted the role of T2 Asp purely as maintenance of the proper structural geometry of the first coordination sphere by means of H‐bonding networks and claim the T3 Glu to be the only proton‐shuttling group in the vicinity of the TNC. Test calculations were run using the CueO rather than CotA structure, to check whether the simulated behavior of the T2 Asp was anomalous feature of CotA laccase. The T2 Asp in CueO showed the same proton‐inactivity.

Simulated pH titrations of T3 Glu in CotA laccase with T2 Asp substituted by Ala, Glu or Asn were later performed as well.^[34d]^ It was concluded that next to structural maintenance of the TNC, the role of T2 Asp was to regulate the T3 Glu by means of long‐range electrostatic interaction. T3 Glu was again stated to be the only proton‐shuttling group close to the TNC. It should be noted however that in all of these simulated pH titrations the enzyme was modeled as a low dielectric continuum, with ϵ=10 and that no explicit solvation shell was used.[^34d^, ^34e^] More rigorous QM/M calculations with explicit water might lead to other results for these residues. Another aspect to take into account is that although the T2 Asp is quite acidic in all CotA structures studied, there are still considerable differences in acidity among them, (Figure 10). For instance, T2 Asp in CotA in the **FR** state (red curve) seems to be about 100 times more alkaline than T2 Asp in CotA with H_2_O_2_ in the TNC (magenta curve). Since none of the structures analyzed in these particular simulated pH titrations is an actual **PI** or **NI**, these titration data do not allow for decisive inferences to the actual pK_a_ of T2 Asp in those catalytic intermediates.

Related to the role of the T2 Asp is the nature of the T2 WD ligand in various intermediates of the catalytic cycle. As set forth throughout section 3, this is a matter of discussion in many of the computational studies. A fundamental problem underlying this discussion is that it is difficult to discriminate between OH^−^ and H_2_O at this coordination site, purely based on the crystal structure.[^31b^, ^32a^, ^46c^, ^63b^] In principle, this issue could be solved by elucidating laccase crystal structures at high resolutions, preferably both in the **RO** and the **FR** state, as it has also been done for AO.[^49^, ^75^] Ideally, this should be accomplished with *Rv*L, because most studies of mechanisms and thermodynamics of laccase catalyzed ORR have used this enzyme. Additionally crystallographic data on high potential laccases would be highly preferable as welL, since it would allow one to more easily relate the structural and hence electronic differences between low and high potential laccases. However, crystallizing large enzymes remains a formidable exercise.

### 4.3 Challenges for simulations

Clarity on the nature of the T2 WD ligand and the role of the second coordination sphere residues may not be related to limited X‐ray resolutions or unavailable crystal structures only. The ambiguities are also related to the systematics by which the computational studies have been carried out. Often the second coordination sphere residues are not explicitly included in the QM region, or they are only included in a fixed protonation state. In the simulations by Yoon and Solomon for instance, T2 Asp is represented by a fixed formate residue and the T2 WD ligand is kept H_2_O throughout the simulation. This is remarkable, given the cooperative mechanism proposed by the same group in the same year, *cf*. Schemes 7 and 8.[^38^, ^42^] An inclusion of such a mechanism in these simulations would have been more in line with expectations. In the later work by Srnec et al. T2 Asp and T3 Glu/Asp representatives were both kept fixed as formate groups and no hypothetical PT affecting the nature of the T2 WD ligand was simulated.^[69]^ Heppner et al. invariably used H_2_O as the T2 WD ligand with a deprotonated formate representing T2 Asp, whereas Siegbahn used a propionic acid H‐bound to crystalline H_2_O.[^37^, ^63b^] Although the simulations performed by Li et al. were exhaustive, the QM systems used in this work neither included a T3 Glu residue, nor a T2 Asp.^[46c]^ A more in‐depth investigation into the influence of the T2 Asp protonation state on the T2 WD ligand could be helpful in resolving this ambiguity.

Arguably, different choices of second coordination sphere residues incorporated in the QM region affect other mechanistic aspects as well. An example would be the details of the **PI** structure. Comparison of the structures obtained or proposed in different theoretical articles shows that deviations from the μ_3_‐1,1,2 coordination of the peroxide ligand, as proposed by Solomon's group, are obtained only if the T2 Asp is omitted or included as a protonated residue.[^31b^, ^38^, ^39^, ^46c^, ^63^] The only exception to this rule is the **PI**(H_2_O; –; μ‐O_2_)_mir_ structure obtained in the *in vacuo* QM/MM simulations by Zhekova et al. (bottom left structure in Scheme 7).^[64]^ The anomalous coordination fashion of the peroxide ligand in this particular structure can possibly be explained by the fact that the enzyme matrix and solvation shell were omitted in this optimization. In addition, the simulations by Zhekova et al. and Siegbahn show that the energetic difference between **PI**(H_2_O; –; μ_3_‐1,1,2‐O_2_) and **PI**(H_2_O; –; μ_3_‐1,1,2‐O_2_)_mir_ is only small if T2 Asp is omitted or represented by a protonated residue.[^63b^, ^64^] Therefore, **PI**(H_2_O; –; μ_3_‐1,1,2‐O_2_) is probably the correct structure, with the T2 Asp carboxylate as the factor facilitating the asymmetric coordination.

Experimental evidence clearly points out the catalytic relevance of the T3 Glu/Asp and T2 Asp.[^31a^, ^39^, ^41^, ^42^] So far, the only computational studies in which PT has been explicitly modeled with one of these second sphere residues explicitly involved are those from Yoon and Solomon and a single calculation in Heppner et al.[^37^, ^38^] In the theoretical analysis of **PI** decay by Yoon and Solomon, the absence of a kinetic solvent effect under basic conditions is attributed to proton transfer occurring only after passing through the transition state at high pH (pH around 7, for *Rv*L or Fet3p). The proton is transferred from the T3 Glu/Asp. As stated earlier, T2 Asp is kept deprotonated in these simulations.^[38]^ Arguing from the typical pK_a_ values ascribed to T2 Asp in *Rv*L and Fet3p, this residue would be significantly deprotonated for pH≥6. Therefore, it is debatable whether a PT event from T3 Glu/Asp to the cleaving O−O bond will actually occur at high pH, even if such an event were strongly favored by a proton‐deficient transition state.^[46c]^ In principle, this could only be possible if the T3 Glu/Asp residue is actually protonated, which seems unlikely under basic conditions. In the calculations by Heppner et al. protons involved in PT were usually modeled as originating from the solvent. In a single calculation however, PT from T3 Glu/Asp to the μ‐OH^−^ in the **NI** intermediate was investigated and found to be 0.6 eV more favorable than protonation from the solvent.^[37]^

The outcomes of both investigations by the Solomon group highlight the need for more elaborate investigations into the roles of key second sphere residues in various stages of the catalytic cycle. In future studies these residues should be included in the QM region and their proton‐shuttling function should be included explicitly in the simulations. It would therefore be interesting to study the pK_a_ of these residues in various intermediates in more detaiL, as this could shed light on the pH optimum observed in laccase catalyzed ORR. For *Rv*L and Fet3p, some pK_a_ values could be determined by means of spectrochemical experiments.[^31a^, ^39^, ^41^, ^42^, ^46a^, ^46b^, ^51^, ^61b^] Such experiments could also be applied to other high potential laccases in various experimentally accessible intermediates, such as **RO** and **NI** in order to obtain empirical data for validation of any computational study into pK_a_ values of second sphere residues. The pK_a_ studies could be combined with a study of the ORR cycle at different physiologically relevant pH values. Such investigations would also be invaluable for determination or verification of pH dependencies of redox potentials belonging to proton‐coupled catalytic steps. These are expected for several elementary steps, but have currently not been published.

Pertaining to the energetic aspects of the simulations, many different computational methodologies have been utilized for calculation of these. For example, modeling the QM region in a protein dielectric medium, QM/MM calculations followed by normal mode analysis and QTCP calculations. In some of the calculations such as those by Yoon and Solomon, only electronic energies are calculated.^[38]^ For a FES, Gibbs energies and true redox potentials are required.

Next to different computational methods, calculated energies have also been calibrated or validated by various methods. To name a few, free energy validation on the kinetics of the reaction by means of the Eyring equation was opted for by Srnec et al.^*[69]*^ Heppner et al. chose to validate their proposed mechanism on kinetic data from ORR catalyzed by *Rv*L and calibrated their free energies to the **RO** to **FR** reaction in *Rv*L.^[37]^ Yet another method was opted for by Siegbahn, who used calibration to an experimentally obtained ORR potential of 0.8 V *vs*. NHE and a formal potential of T1 Cu^II^/Cu^I^ of CueO.^[63b]^ In several simulations, the energetics of the proposed mechanism have been validated by associated kinetic data, using for instance steady state or Marcus theory. Such validation methodologies are worth copying, provided that the kinetic data originates from the same MCO as the crystal structure on which the optimizations were run. This is not the case for the simulations by Heppner et al., as these investigators based their calculations on the CueO crystal structure and related their mechanism to ORR kinetics in *Rv*L.^[37]^ It has been shown that the kinetic details of similar elementary steps differ among different laccases.[^27f^, ^30c^] The calibration method applied by Siegbahn seems straightforward, however, the work of Li et al. has demonstrated that the T1 Cu^II^/Cu^I^ redox couple varies throughout the catalytic cycle.^[46c]^ Consequently, using a standard redox potential for this redox couple may be an oversimplification. Yet it is the redox potential of the T1 site that governs whether the kinetics of IET are fast or rate limiting, and whether the ORR can proceed at a low overpotential.

Free energy calibration by means of kinetic models is a good validation method for any elementary step for which kinetics are determined experimentally. This is not the case for all steps and most kinetic data comes from *Rv*L, whereas simulations are based on CueO or *Tv*L, two MCOs likely to be featured by other kinetic details.^[30c]^ Therefore it may be meaningful to calibrate any computations of ET or PCET events on the **RO** to **FR** reaction for which oxidation potentials are available. This will give confidence in the validity of potentials further down the catalytic cycle and which are experimentally inaccessible. Note that such an approach differs from the ‘resting calibrated’ methodology of Heppner et al. They only used the **RO** to **FR** reaction to get an indication of the overestimations in their free energy calculations. The overestimation found for **RO** to **FR** was subsequently assumed to be applicable to all other catalytic steps. It is difficult to verify whether this is true.

### 4.4 Elucidation of a preliminary FES for the laccase catalyzed ORR

Construction of the FES of laccase catalyzed ORR requires the mechanism to be known to the level of each elementary step. Currently, only the structures of spectrochemically observable key intermediates are well defined. Voltammetric methodologies turn out to be unsuitable for obtaining additional mechanistic information. Since no individual intermediates can be observed voltammetrically, the thermodynamics required for a FES cannot be determined either by this type of experiments. Therefore, further mechanistic insights are to be obtained from computational modeling, in which the key intermediates could serve as the backbone for filling out the gaps in the mechanism.

**Figure 11:**
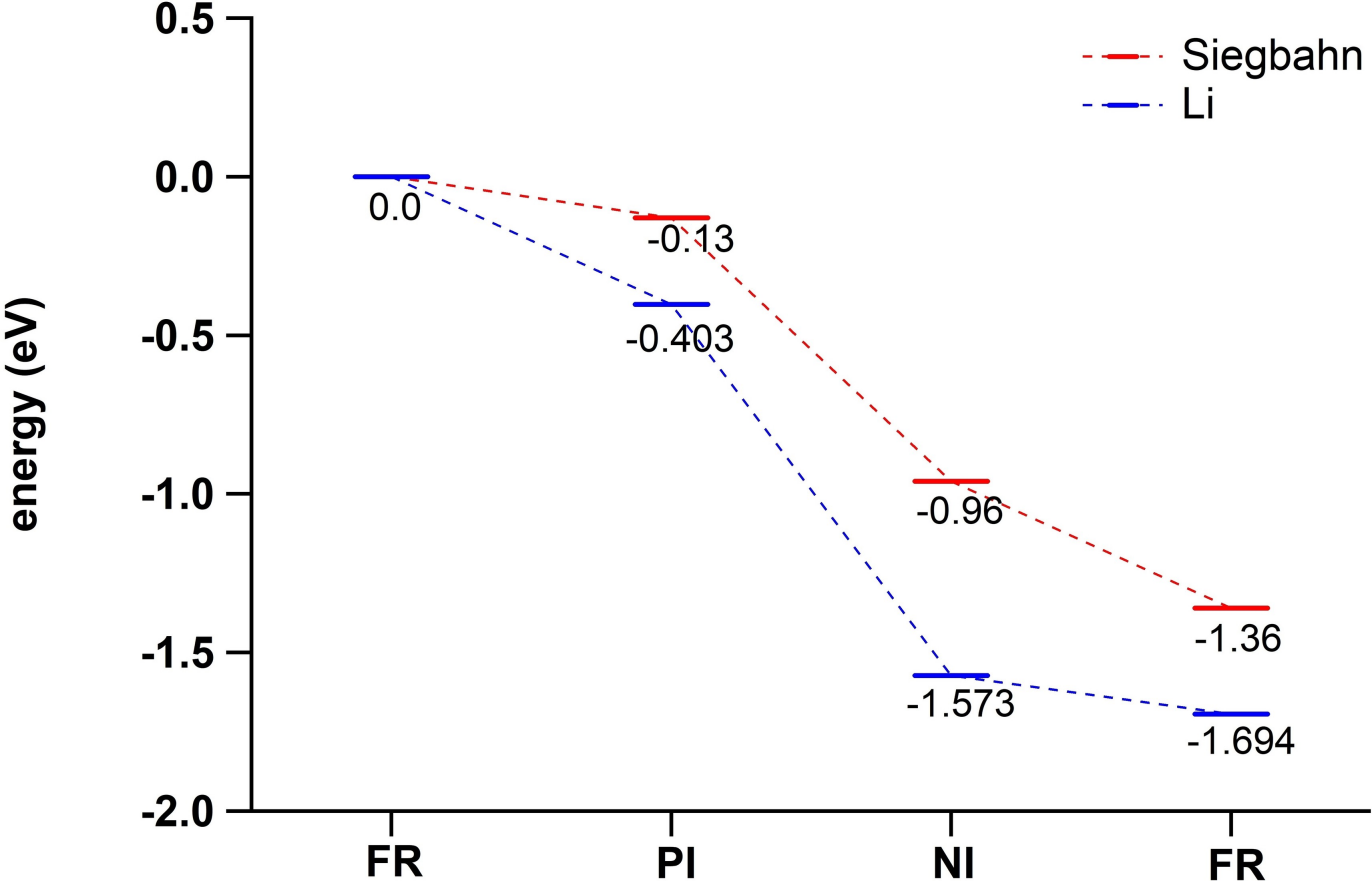
Free energy landscape of the Lacasse key intermediates **FR**, **PI** and **NI** based on the work of Siegbahn (red, 10 % HF) and Li et al. at pH 7. Energies are scaled versus the reduction potential of 0.46 V for Cu T1.

For this purpose, QM/MM methodology with two QM regions and a solvation shell of explicit water would be most ideaL, preferably based on the crystal structure of an actual laccase. The first QM region should incorporate the T1 complex, the other should include the first coordination sphere of the TNC Cu ions including the T3 Glu/Asp and T2 residues. Such a model with two QM regions would be most suitable for calculations of redox potentials associated with IET from the T1 site to the TNC. Inclusion of T3 Glu/Asp and T2 Asp residues is required for gaining more insight in the proton mediating, ligand‐, and geometry‐determining roles of these groups in various elementary steps, as well as their influence on the thermodynamics of these reactions. This might be of help in elevating the differences in structures and mechanisms proposed in the currently available body of computational studies. Inclusion of these residues in the QM region also allows for accurate modeling and comparison of explicit PT events at the quantum mechanical leveL, which as of yet has not been done extensively. In addition, the reaction could be simulated under different pH values so as to obtain an improved understanding of the non‐Nernstian pH‐dependent catalytic activity of laccase for the ORR. To be of use for FES formation, the calculated energies should be Gibbs energy and not electronic energies. Calculating free energies requires conformational sampling to account for enthalpic and entropic contributions next to the electronic energy contribution. Pertaining to the validation of the results, various proposals can be made. In the first place, reactive pathways can be assayed by validating the associated free energies with kinetic data, if these are available. This validation method requires the kinetic data to be associated to ORR catalysis in the same laccase as the crystal structures of the laccase on which the geometry optimizations are based. Secondly, calculations of redox potentials could be validated by also simulating anaerobic reduction of the enzyme and comparing the results to experimentally obtained data. As a matter of fact, the empirical basis for this kind of calibration is rather feeble, because TNC redox data are only documented for *Rv*L and *Pv*L; besides, these data comprise formal potentials only. The strength of the validation method outlined here could be improved by repeating the redox titrations by Reinhammar and Vänngård on both high and low potential laccases over a physiologically relevant pH‐range in order to obviate the limitations of formal potentials. At present only a very limited amount of data is available that is based on calculated Gibbs energies rather than electronic energies. A crude FES can be drawn on basis of the results by Li and Siegbahn, which are shown in Figure 11. At the conditions chosen in both studies the equilibrium potential of the ORR is 0.82 V (pH 7), while a potential of 0.46 V was selected that is typical for a low potential laccase T1 site. Under these conditions a full oxygen reduction cycle at the TNC is exothermic by 1.44 eV. Most of this energy gain is achieved by O−O bond cleavage upon O_2_ binding to **FR** to produce **NI**. This especially holds in the studies by Li et al., where binding and cleavage of O_2_ is exothermic by −1.57 eV, and suggests that the reduction steps from **NI** to **FR** must occur more or less on a flat potential energy surface. Although this FES and sequence of reaction steps may explain well how the ORR occurs in low potential laccases such as CueO, it does not explain how ORR can occur with a very low overpotential when mediated by high potential laccases, where the redox potential of the T1 site is close to 0.8 V (see Table 4). Under such conditions, a full oxygen reduction cycle can only be slightly exothermic. Within such a system a decrease in free energy upon cleavage of the O−O bond to achieve an **NI** state with a free Gibbs energy of roughly −1 eV would represent a major thermodynamic sink. Theoretical studies that allow for a more detailed and accurate description of the potential energy surface of high potential laccases are necessary in order to explain why laccase is such an efficient catalyst for the oxygen reduction reaction.

**Table 4:** E_1/2_ values for CV experiments on high potential laccases from Trametes hirsuta (*Th*L) and from Trametes versicolor (*Tv*L) immobilized on coated Au electrodes. All results were obtained at or close to 298 K, at pH 6.5.

Method	Enzyme	E_1/2,low_	E_1/2,high_	Ref.
		[mV *vs*. NHE]	[mV *vs*. NHE]	
I	*Th*L	405	none	[55c]
II	*Th*L	394± 3	778± 4^ii^	[27e]
II	*Tv*L	408± 4	784± 3^ii^	[27e]

I) Anaerobic CV with capillary Au electrode in a buffered solution containing 10 mg/mL laccase without redox mediators. Scan rate was 10 mV/s. Potential window ranged from 80–103 mV vs. NHE. II) Anaerobic CV with enzyme entrapped within solid, electrochemically inert TBMPC polystyrene cross‐linked with 1 % (w/v) divinylbenzene coating on Au electrode. Scan rate was 50 mV/s. Potential window ranged from 50–950 mV vs. NHE.

## 5 Conclusion

Even though laccase is one of the best electrocatalysts reported thus far, we do not yet fully understand how the enzyme is capable of catalyzing the oxygen reduction reaction efficiently. Over the last decades a tremendous amount of research has been performed on laccase systems, and an overall mechanism can be proposed which includes the intermediates **FR**, **PI** and **NI**. Only the slow enzyme activation step from **RO** to **FR** can be probed experimentally, which means that thermodynamic values involving the other intermediates has to come from theory.

Several reports have been published, which are summarized here, providing a general consensus concerning the mechanism wherein the ORR occurs. However, the devil is in the details. There is still debate on the precise mechanistic pathway between **PI** to **NI** and **NI** to **FR**. The inclusion of the Glu/Asp and T2 Asp residues are expected to have a major influence on the computational results. Moreover, free energies would need to be computed consistently to allow for the construction of an accurate FES. Overcoming these challenges should be a next goal for future computational research to elucidate the FES of laccase catalyzed ORR in more detaiL, and contribute to the development of newer and better catalysts that we need for our future energy infrastructure.

## Conflict of interest

The authors declare no conflict of interest.

## Biographical Information

*Daan den Boer is a Ph.D. candidate under supervision of Dr. Dennis Hetterscheid at Leiden university. His current research focuses on the design principles of homogeneous and bio‐inspired redox catalysts. The research especially focuses on the influence of the electronic structure of the catalyst and interactions between the catalyst and the electrode surface*.

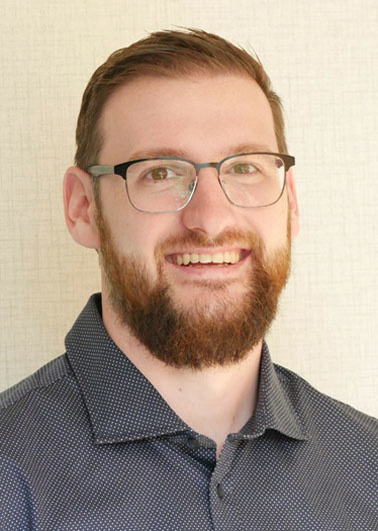

## Biographical Information

*Hendrik C. de Heer received his B.Sc. in chemistry from Leiden University in 2020. This review is heavily based on his B.Sc. thesis. As a Master student he continued working under the supervision of Dr. Hetterscheid at Leiden University. His current research interest is the homogeneous catalysis of the dihydrogen peroxide reduction reaction by mononuclear copper complexes*.

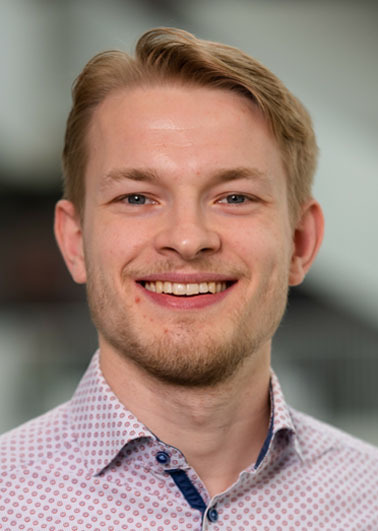

## Biographical Information

*Francesco Buda is an assistant professor at Leiden University since 2001. After obtaining a Ph.D. from the International School for Advanced Studies, Italy, in 1989, he held Postdoctoral positions at Ohio State University and IBM Zurich Research Laboratory. He was also a Researcher of the INFM, Scuola Normale Superiore, Pisa, Italy. He has a record of accomplishments in ab‐initio and quantum‐classical molecular dynamics simulations applied to catalytic reactions and photoactive proteins. His research interests include natural and artificial photosynthesis to translate the insight from simulations into the design of molecular and heterogeneous complexes for solar to fuel conversion*.

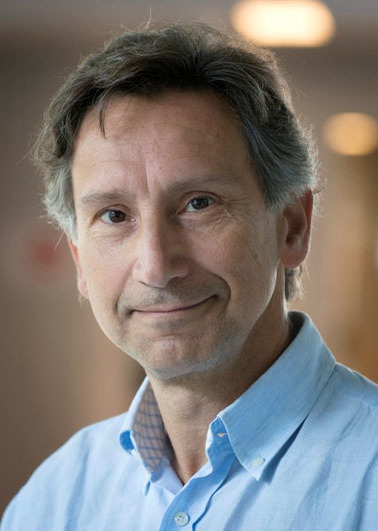

## Biographical Information

*Dennis is an Associate Professor at Leiden University. Previously he has obtained his Ph.D. from the Radboud University of Nijmegen and was a postdoctoral fellow at the Massachusetts Institute of Technology (MIT) and the University of Amsterdam. The activities of his research group at the Leiden Institute of Chemistry are mainly geared towards understanding and mimicking bioinorganic multi‐electron processes that are relevant to a future energy infrastructure and circular economy. Of particular interest are the oxygen evolution and oxygen reduction reactions, reactions relevant to denitrification, the activation of carbon monoxide and dinitrogen, and the electrochemical synthesis of useful organic molecules*.

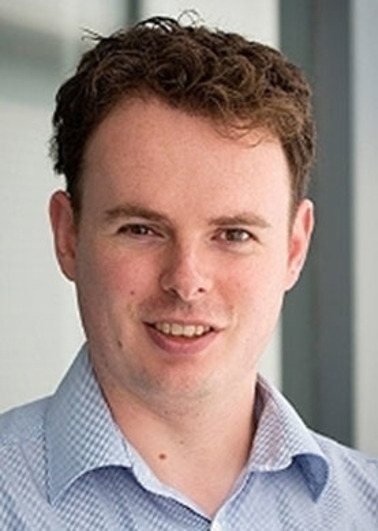

## Footnotes

n1 NB: onset potentials for both electrodes were different: laccase‐wiring electrode had onset potential of 20 mV. Pt‐electrode had onset potential of 120 mV.

n2 Note that Srnec et al. refer to these residues as Asp‐82 and Glu‐453, whereas Bento et al. state that the T2 Asp residue in CueO is Asp‐112 and the Glu residue is Glu‐506. Siegbahn also mentions Asp‐112. Remarkably, Siegbahn used the same PDB structure as Srnec et al. From the figures in the articles mentioned here, it is apparent that T2 Asp is meant, although it is referred to with different numbers. Comparison of the primary structure in the PDB of the structure in question (PDB code is 1KV7) yields Phe at position 112 and a sequence of 488 residues. This implies that Glu‐506 does not occur. Residue 453 is Glu, in line with Srnec et al. This seems to be an anomalous feature in the literature.
